# Superstructured Biomaterials Formed by Exchange Dynamics and Host–Guest Interactions in Supramolecular Polymers

**DOI:** 10.1002/advs.202004042

**Published:** 2021-02-22

**Authors:** Alexandra N. Edelbrock, Tristan D. Clemons, Stacey M. Chin, Joshua J. W. Roan, Eric P. Bruckner, Zaida Álvarez, Jack F. Edelbrock, Kristen S. Wek, Samuel I. Stupp

**Affiliations:** ^1^ Department of Biomedical Engineering Northwestern University Evanston IL 60208 USA; ^2^ Simpson Querrey Institute Northwestern University Chicago IL 60611 USA; ^3^ Department of Chemistry Northwestern University Evanston IL 60208 USA; ^4^ Department of Materials Science and Engineering Northwestern University Evanston IL 60208 USA; ^5^ Department of Medicine Northwestern University Chicago IL 60611 USA

**Keywords:** 3D printing, adamantane, biomaterials, cyclodextrin, host–guest, peptide amphiphile, superstructure

## Abstract

Dynamic and reversible assembly of molecules is ubiquitous in the hierarchical superstructures of living systems and plays a key role in cellular functions. Recent work from the laboratory reported on the reversible formation of such superstructures in systems of peptide amphiphiles conjugated to oligonucleotides and electrostatically complimentary peptide sequences. Here, a supramolecular system is reported upon where exchange dynamics and host–guest interactions between *β*‐cyclodextrin and adamantane on peptide amphiphiles lead to superstructure formation. Superstructure formation with bundled nanoribbons generates a mechanically robust hydrogel with a highly porous architecture that can be 3D printed. Functionalization of the porous superstructured material with a biological signal results in a matrix with significant in vitro bioactivity toward neurons that could be used as a supramolecular model to design novel biomaterials.

## Graphical abstract

In this work, exchange dynamics and host–guest interactions between *β*‐cyclodextrin and adamantane on peptide amphiphiles lead to hierarchical superstructure formation. This superstructure leads to mechanically robust, highly porous hydrogels amenable to 3D printing. Functionalization of the porous superstructured material with a biological signal results in a matrix with significant in vitro bioactivity toward neurons.

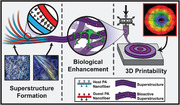

## 1 Introduction

Reversible hierarchical self‐assembly of molecules, through multiple orthogonal interactions have been harnessed by living systems to control the formation of structures such as protein assemblies, cellular membranes, and cytoskeletal filaments and countless other examples.^[^ ^1^, ^2^, ^3^, ^4^ ^]^ These highly organized but dynamic structures, held together by non‐covalent interactions, play critical roles in the regulation of life processes.^[^ ^5^, ^6^ ^]^ The non‐covalent bonds enable transient complexation among molecules of interest, thus allowing reversible and dynamic functionality in the hierarchical structures of living matter.^[^ ^7^ ^]^ The ability to emulate this dynamic hierarchical self‐assembly in synthetic systems has proven to be challenging, especially to trigger the reversible assembly of molecules.

Recent work from our laboratory reported the formation of hierarchical superstructures through dynamic exchange of molecules in supramolecular nanofibers.^[^ ^8^ ^]^ The supramolecular structures were assemblies containing peptide amphiphiles (PAs) conjugated to complementary oligonucleotides. In these systems, Watson–Crick pairing was the driving force that built the superstructures via dynamic exchange of molecules. This work also showed that PAs designed with oppositely charged peptide domains can generate superstructures by a similar mechanism. This surprising result is highly dependent on the dynamic exchange of molecules among the filamentous assemblies observed in previous work by Meijer and coworkers and Stupp and coworkers.^[^ ^9^, ^10^ ^]^ However in the most recent work^[^ ^8^ ^]^ we found that dynamic exchange driven by the establishment of enthalpically driven interactions can be used to build hierarchical superstructures. Another interesting feature of these dynamic systems was the finding that superstructure formation was a reversible phenomenon. Reversibility was achieved by the addition of oligonucleotide displacement strands which out‐competed the Watson–Crick pairing, forming inter‐fiber crosslinks or by pH changes that diminished electrostatic interactions.^[^ ^8^ ^]^

In very recent work, we have demonstrated the importance of the *β*‐sheet peptide sequence in PA molecules in preventing their escape from the assemblies given the greater internal cohesion.^[^ ^11^ ^]^ In these systems, the mixing of two PA assemblies with opposite charge did not allow hierarchical superstructures to form. However, when the PA molecules do not contain *β*‐sheet hydrogen bonding, molecular escape is no longer inhibited, and this enables the self‐assembly into hierarchical structures. In earlier work, coarse‐grained simulations suggested that when the strength of non‐covalent interactions within PA nanofibers is low enough to allow molecules to escape, reorganization into superstructures is possible provided there is a driving force to lower free energy by enhancing inter‐assembly interactions. These studies demonstrate the opportunity to create dynamic soft matter by using the remarkable dynamics of supramolecular assemblies combined with molecular design that directs the system to engage in strong interactions.

In this work, we investigate the possibility of using dynamic molecular exchange to form bioactive hierarchical superstructures based on PA molecules designed to engage host–guest interactions. The host–guest complex between *β*‐cyclodextrin and adamantane is well known to form a strong, reversible non‐covalent interaction.^[^ ^12^, ^13^ ^]^ Our laboratory used it previously to demonstrate dynamic bioactivity with adamantane conjugated to the integrin binding peptide epitope RGDS and alginate substrates functionalized with cyclodextrin.^[^ ^14^ ^]^ This host–guest interaction has been well studied for a range of biomedical applications including stimuli‐responsive hydrogels,^[^ ^15^ ^]^ biosensing,^[^ ^16^, ^17^ ^]^ drug release,^[^ ^18^ ^]^ 3D printing,^[^ ^19^ ^]^ and scaffold functionalization.^[^ ^20^ ^]^ The Harada group has extensively studied host–guest interactions based primarily on cyclodextrin inclusion complexes to develop stimuli responsive actuating materials^[^ ^21^, ^22^ ^]^ and also systems with self‐healing adhesive properties.^[^ ^23^ ^]^ Mata and coworkers recently explored the combination of positively charged PAs with a strong *β*‐sheet forming peptide region and host–guest moieties.^[^ ^24^ ^]^ Their study established the use of host–guest interactions as a viable tool to enhance mechanical properties of PA hydrogels, effectively functioning as crosslinks between fibers. PA hydrogels are now well established as supramolecular biomaterials that can be designed to be highly bioactive and effectively signal cells to promote tissue regeneration and other biomedical targets.^[^ ^25^, ^26^, ^27^, ^28^, ^29^ ^]^ One recent example with remarkable bioactivity was the functionalization of a PA molecule with a cyclic peptide that mimics the important growth factor in neural biology known as brain derived neurotrophic factor (BDNF). These supramolecular nanofibers successfully activated BDNF receptors and increased functional neuronal maturation and cell infiltration on PA scaffolds in vitro.^[^ ^30^ ^]^

Also, the ability to coassemble PA molecules in the supramolecular polymers they form provides potential to display multiple functional groups at tunable densities. We report here on a bioactive PA system designed to exhibit dynamic exchange of monomers and also functionalized with *β*‐cyclodextrin and adamantane moieties as a strategy to create reversible hierarchical superstructures. To introduce bioactivity in this system, we utilized the BDNF mimetic signal and studied in vitro its biological activity in the presence of neurons. This combination of dynamic crosslinking and potential bioactivity could provide a 3D printing platform for spatially defined templates for populations of cells that can simulate the complexity of neural tissue for in vitro assays. Furthermore, in this work we pursued this objective without the need for additive molecules which may alter bioactivity of the materials used. Extruding neuronal cells is particularly challenging since the bioink carrier must be of appropriate softness to avoid shearing them while subsequently maintaining an appropriate stiffness so the construct maintains its shape. In this work, we have investigated systems that can combine bioactive chemical signaling and dynamic hierarchical superstructure formation to construct 3D printed self‐supporting cell laden hydrogels.

## 2 Results and Discussion

### 2.1 Design and Characterization of Host–Guest Modified Peptide Amphiphile Supramolecular Polymers

**Figure 1:**
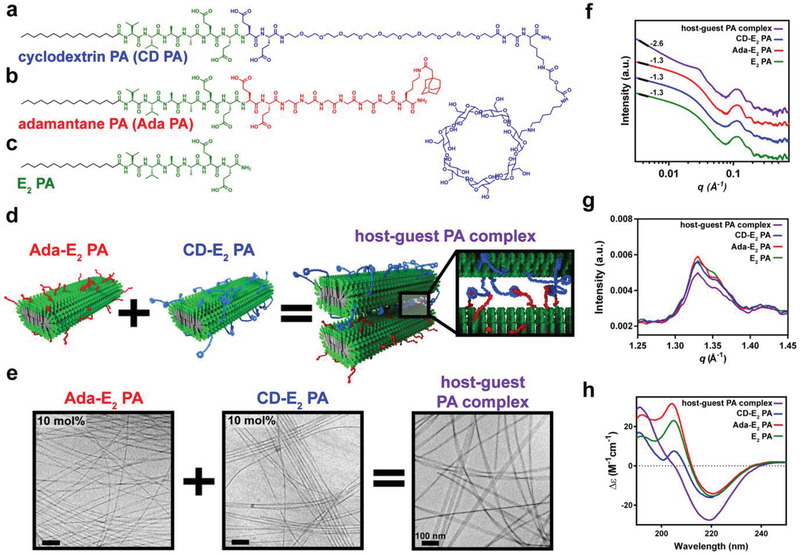
Design and characterization of host–guest modified peptide amphiphiles (PAs). a–c) Chemical structures of a) the cyclodextrin PA (CD PA), b) the adamantane PA (Ada PA), and c) E_2_ PA. d) Schematic representation of the supramolecular polymer formed by the Ada‐E_2_ PA, the CD‐E_2_ PA, and their mixture forming host–guest complexes. e) Cryogenic transmission electron micrographs of Ada‐E_2_ PA, CD‐E_2_ PA, and their mixture. f) Small angle x‐ray scattering patterns, g) wide angle x‐ray scattering patterns, and h) circular dichroism spectra corresponding to the mixture of CD‐E_2_ PA and Ada‐E_2_ PA (purple), CD‐E_2_ PA (blue), Ada‐E_2_ PA (red), and E_2_ PA (green). The CD PA and Ada PA were each coassembled at 10 mol% with the E_2_ PA separately to form the CD‐E_2_ PA and Ada‐E_2_ PA, respectively. These materials were then mixed 1:1 by volume to make the host–guest PA complex for all experiments.

We synthesized two new PA molecules functionalized to display either a *β*‐cyclodextrin host or an adamantane guest. A major consideration in the design of both PAs was to position the host and guest moieties away from the surface of the PA nanofibers and most importantly to promote dynamic molecular exchange among nanofibers to spontaneously create hierarchical structures. A polyethylene glycol (PEG_10_) spacer was chosen to link *β*‐cyclodextrin to PA molecules based on its hydrophilicity and molecular flexibility (**Figure** **1**a).^[^ ^8^, ^31^, ^32^, ^33^ ^]^ Since adamantane is much more hydrophobic, six glycine residues were used as a linker to minimize this moiety folding back and interacting with the hydrophobic core of PA nanofibers (Figure 1b). Both the cyclodextrin PA (CD PA) and adamantane PA (Ada PA) had the same chemical sequence conjugated to the linker and host or guest moieties, namely a 16‐carbon alkyl tail followed by the peptide sequence V_2_A_2_E_4._ The E residues in the sequence ensured aqueous solubility for the 100 mol% Ada PA and CD PA molecules.^[^ ^34^ ^]^ Furthermore, we hypothesized that the high charge of the PA molecules provided by the E_4_ sequence would facilitate dynamic exchange due to repulsive forces and thus enable reorganization into hierarchical assemblies.^[^ ^11^ ^]^ To prevent overcrowding among host and guest moieties and improve their display on nanofiber surfaces, the CD PA and Ada PA were coassembled with a diluent PA with the sequence C_16_V_2_A_2_E_2_ PA (E_2_ PA) (Figure 1c). An E_2_ PA molecule was chosen because it is known to form robust nanoribbons and can be coassembled with many other PA sequences displaying bioactive epitopes.^[^ ^35^ ^]^ The CD PA and Ada PA solutions were prepared separately with or without E_2_ PA in 125 × 10^−3^ m NaCl and 3 × 10^−3^ m KCl, annealed at 80°C for 30 min, and slowly cooled to drive them to the thermodynamically favored formation of long fibers.^[^ ^36^ ^]^ These two solutions were then mixed 1:1 by volume to allow the host–guest complex to form among nanofibers (Figure 1d). The molecular schematic in Figure 1d illustrates formation of the host–guest complex between the two supramolecular coassemblies before any molecular exchange would enable the formation of superstructures.

Cryogenic transmission electron microscopy (Cryo‐TEM) was used to visualize the host‐ and guest‐modified supramolecular polymers coassembled with the E_2_PA at compositions varying from 0 to 100 mol%. Neither 100 mol% CD PA nor 100 mol% Ada PA exhibited formation of nanofibers through 1D supramolecular polymerization (Figure S1a, Supporting Information). Dynamic light scattering (DLS) data indicated that the Ada PA formed micelle‐like structures with a diameter distribution centered at 9 nm, with both PAs also forming structures greater than 300 nm in diameter suggesting the presence of some larger PA aggregates (Figure S1b, Supporting Information). When E_2_ PA was added to the CD PA and Ada PA, the formation of long 1D nanoribbons with monodisperse widths was observed using cryo‐TEM in 10 mol% coassemblies (10 mol% adamantane or cyclodextrin PA and 90 mol% E_2_ PA) (Figure S2, Supporting Information). Furthermore, it was evident that coassembly ratios above 75 mol% of the functionalized PA significantly disrupted fiber formation as observed by conventional TEM (Figure S3, Supporting Information). Therefore, a 10 mol% coassembly ratio of the CD PA and Ada PA with E_2_ PA was used for all subsequent experiments and we refer to these supramolecular polymers as CD‐E_2_ PA and Ada‐E_2_ PA, respectively. Cryo‐TEM of CD‐E_2_ PA, Ada‐E_2_ PA, and the mixture of both supramolecular polymers revealed the formation of ribbon‐like structures (Figure 1e). The approximate average width of the CD‐E_2_ PA and Ada‐E_2_ PA assemblies was 16 ± 3 nm and 17 ± 3 nm, respectively, and the mixture of both was found to form wider ribbons measuring on average 26 ± 8 nm in width (Figure S4, Supporting Information). We hypothesized that the observed increase in fiber width could be due to host–guest interactions among the ribbon‐shaped supramolecular polymers. This was further investigated using synchrotron small angle x‐ray scattering (SAXS, Figure 1f). The E_2_ PA, CD‐E_2_ PA, and Ada‐E_2_ PA exhibited a slope of ‐1.3 in the Guinier region consistent with the presence of thin ribbon structures.^[^ ^37^ ^]^ Consistent with cryo‐TEM observations, the mixture of host and guest PAs had a slope of ‐2.6, indicative of wider ribbons which presumably form as a result of complex formation and bundling of the supramolecular polymers.^[^ ^38^ ^]^ Furthermore, there was a clear emergence of a Bragg peak at a *q* value of 0.0254 Å^–1^ corresponding to a d‐spacing of approximately 24.7 nm, similar to the average dimension observed in Cryo‐TEM images, consistent with the formation of a uniform hierarchical assembly resulting from this interaction. Wide‐angle synchrotron x‐ray scattering (WAXS) measurements confirmed the presence of *β*‐sheets in the interior of the assemblies with a peak at approximately 1.33 Å^–1^ corresponding to a d‐spacing of 4.72 Å in all cases except for the non‐ribbon forming 100 mol% CD PA and Ada PA samples (Figure 1g; Figure S5, Supporting Information). This d‐spacing is considered typical of *β*‐sheet secondary structures reported for many amyloid and peptide fibrillar assemblies.^[^ ^38^, ^39^, ^40^ ^]^ Circular dichroism spectroscopy further confirmed the presence of *β*‐sheet secondary structure in the E_2_ PA, Ada‐E_2_ PA, CD‐E_2_ PA, and also within the host–guest PA complex sample (Figure 1h). An apparent red shift in the spectra was observed relative to those in planar *β*‐sheets with a maximum at 195 nm and a minimum at 218 nm.^[^ ^41^ ^]^ This red shift has been previously attributed to an increase in the twist within anti‐parallel *β*‐sheets and we propose this is also occurring in our system.^[^ ^42^, ^43^, ^44^ ^]^ Finally, Fourier‐transform infrared spectroscopy (FTIR) also supported the presence of *β*‐sheet secondary structure, revealing the expected absorbance peak at approximately 1625 cm^–1^ for all structures corresponding to the characteristic amide I band (Figure S6, Supporting Information).^[^ ^45^ ^]^ These observations establish the incorporation of the host–guest moieties in the PA supramolecular polymers investigated here does not disrupt supramolecular assembly.

### 2.2 Characterization of Host–Guest Modified Peptide Amphiphile Superstructure Formation

To assess the extent of host–guest interactions, proton nuclear magnetic resonance (^1^H‐NMR) spectra were obtained to determine the binding isotherms of the CD‐E_2_ PA and Ada‐E_2_ PA (Figures S7–S9, Supporting Information). Since hydrogel formation results in extensive broadening of NMR signals, CD‐E_2_ PA was titrated with soluble 1‐adamantaneacetic acid and Ada‐E_2_ PA was titrated with *β*‐cyclodextrin. The binding isotherms showed that both the CD‐E_2_ PA and Ada‐E_2_ PA follow the stoichiometry of a 1:1 host–guest system with association constants (K_*α*_) of 434 M^–1^ and 3378 M^–1^, respectively. This is consistent with previously reported values in literature, which report association constants ranging from 10^2^ to 10^4^ m ^–1^ for the interaction of *β*‐cyclodextrin with adamantane moieties when incorporated into supramolecular gels.^[^ ^24^, ^46^ ^]^ The difference in association constants between the CD‐E_2_ PA and Ada‐E_2_ PA is likely due to the changes in spacer length and conformational flexibility, where for example, the PA tethered adamantane is likely to be more hydrophobic coupled through an amide bond to a hydrophobic glycine spacer relative to the 1‐admantaneacetic acid used with the CD‐E_2_ PA. Importantly, 1:1 stoichiometry of the binding was observed, which is critical for inter‐fiber crosslinking.

**Figure 2:**
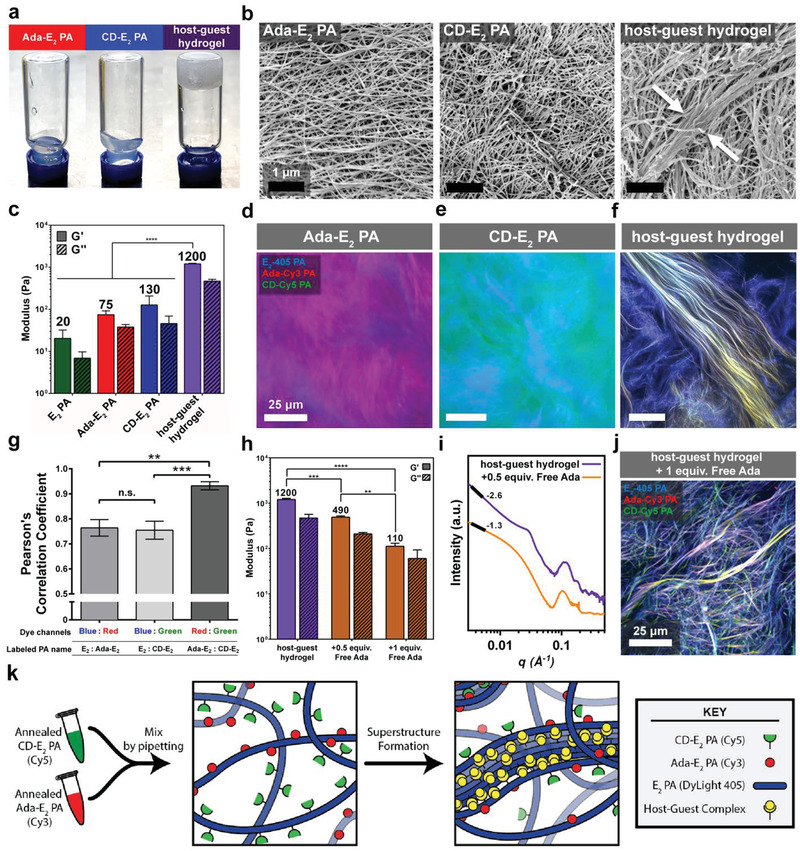
Characterization of host–guest superstructure formation. a) Photographs of solutions in inverted vials of Ada‐E_2_ PA, CD‐E_2_ PA, and of the self‐supporting hydrogel formed by their mixture (host–guest hydrogel). b) Scanning electron micrographs showing nanoribbons in gelled Ada‐E_2_ PA, CD‐E_2_ PA, and the characteristic superstructures (between white arrows) formed in the host–guest hydrogel. c) Storage (G′) and loss (G″) moduli of (left‐right) the E_2_ PA, Ada‐E_2_ PA, CD‐E_2_ PA, and the host–guest hydrogel. Confocal optical micrographs of Ada‐E_2_ PA labeled with d) Cy3 dye (red), the CD‐E_2_ PA labeled with e) Cy5 dye (green), and f) their mixture to form the host–guest hydrogel (all samples were coassembled with the E_2_ PA labeled with DyLight 405 dye (blue)). g) Pearson's correlation coefficient analysis quantifying colocalization of the blue and red channels (left), blue and green channels (middle) and the red and green channels (right) from micrographs of the host–guest hydrogel. h) Storage (G′) and loss (G″) moduli of (left‐right) the host–guest hydrogel, the host–guest hydrogel plus 0.5 equivalents (equiv.) of free adamantane (Free Ada), and the host–guest hydrogel plus 1 equiv. Free Ada. i) Small angle x‐ray scattering curves of the host–guest hydrogel and the host–guest hydrogel plus 0.5 equiv. of Free Ada. j) Confocal micrograph using dye labels described in (d) after the addition of 1 equivalent of Free Ada to the host–guest hydrogel. k) Schematic representation of superstructure formation resulting from molecular exchange of the host and guest functionalized peptide amphiphiles. Upon mixing of the Ada‐E_2_ PA (red) and CD‐E_2_ PA (green), redistribution of host and guest functionalized molecules through molecular exchange results in the localized enrichment of these functionalized molecules to form hierarchical superstructures. **p < 0.01, ***p < 0.001, and ****p < 0.0001 LSD test (c) (*n* = 3), (g) (*n* = 10), (h) (*n* = 2).

When the CD‐E_2_ PA and Ada‐E_2_ PA were mixed, formation of a soft gel occurred within a minute of pipetting as a result of host–guest crosslinking among PA nanoribbons. While individual PAs formed clear solutions, a stable and opaque self‐supporting gel, the host–guest hydrogel, was formed upon mixing (**Figure** **2**a). Scanning electron microscopy (SEM) was used to study the morphology of the material formed and its individual components (Figure 2b) (all samples were gelled with divalent cations prior to the required processing for SEM imaging). The CD‐E_2_ PA and Ada‐E_2_ PA samples contained similar homogenous supramolecular polymer structures whereas the host–guest hydrogel revealed large bundles (≈1 µm in size) of the nanostructures across the surface of the gel. Differences were also observed in the bulk rheological properties of these systems. Storage (G′) and loss (G″) moduli were measured for the individual components and the host–guest hydrogel (see Figure 2c). Gels of E_2_ PA, Ada‐E_2_ PA, and CD‐E_2_ PA had storage moduli of 20 ± 12 pascals (Pa), 75 ± 17 Pa, and 130 ± 81 Pa, respectively. However, the storage modulus was observed to dramatically increase to 1200 ± 34 Pa when the Ada‐E_2_ PA and CD‐E_2_ PA were mixed 1:1 by volume. When a higher concentration of Ada‐PA (25 mol% Ada‐PA) relative to the CD‐PA (10 mol% CD‐PA) was investigated, an increase in storage modulus was observed relative to the 1:1 stoichiometric ratio (Figure S10, Supporting Information). This result is consistent with previous studies on a similar system, where the increase in guest concentration relative to the host, facilitated multiple polymer chain entanglements joined by several host–guest complexes which dominated the moduli of the hydrogel.^[^ ^46^ ^]^ To test the host–guest hydrogel's recovery properties, a high strain was applied to fracture the gel which caused G′ to drop below the value measured for G″ (Figure S11, Supporting Information). When the high strain was removed, the host‐guest hydrogel returned to its original values of G′ and G″. We hypothesize that this reversible change in rheological properties indicates the material possesses self‐healing capabilities mediated by the reversible host–guest interactions among the supramolecular polymers. Based on our observation of large bundles of nanoscale ribbons in the host–guest hydrogel, we hypothesized that the intense dynamic event previously observed by our laboratory has occurred in the system upon mixing.^[^ ^8^, ^11^ ^]^ This interesting phenomenon involves the spatial relocation across long distances (possibly microns) of monomers (or monomer clusters) containing either host or guest moieties in order to concentrate and optimize the number of the highly favorable host–guest contacts. We refer from now on to the observed large bundles of supramolecular polymers as a “superstructure” whose formation is hypothesized to occur through this dynamic mechanism.

To test our hypothesis that exchange dynamics in these supramolecular systems contributed superstructure formation, we designed and synthesized additional host–guest modified peptide amphiphiles with stronger intermolecular cohesion, containing two additional valine residues which was shown in previous work to increase the strength of the *β*‐sheet.^[^ ^42^ ^]^ One glutamic acid residue was also removed to lower the assembly's charge density which obviously increases further the cohesive energy of the supramolecular polymers. The objective of these modifications in the structure of PAs was to reduce the propensity for molecular exchange consistent with previous work.^[^ ^8^, ^11^ ^]^ These new molecules formed nanoribbons as observed by cryo‐TEM and all contain evidence of internal *β*‐sheet character as observed by CD (Figure S12, Supporting Information). Interestingly, we did not observe significant hierarchical superstructure formation within the mixture of these control molecules as demonstrated by SEM micrographs (Figure S13a, Supporting Information). Bundle formation was significantly reduced with only small bundled structures of approximately 150 nm in width observed as opposed to ≈1 µm in the supramolecular polymers where we indeed observed dynamic exchange. Providing further evidence for our suggestion, the storage modulus of these mixtures was not enhanced relative to that of individual cyclodextrin and adamantane peptide amphiphile supramolecular polymers indicating that the stronger *β*‐sheet and reduced charge indeed inhibit molecular exchange (see Figure S13b,c, Supporting Information).

To study the superstructured material bundling phenomena further, confocal microscopy was used to allow us to probe the internal structure of the gels formed. To enable visualization, the PAs were modified with fluorophores, the E_2_ PA was modified with DyLight 405 (Blue) while the Ada PA and CD PA were modified with cyanine 3 (Cy3, Red) and cyanine 5 (Cy5, Green), respectively. Samples of the CD‐E_2_ PA and Ada‐E_2_ PA with the fluorophores were both liquid in nature and their micrographs revealed isotropic solutions with small aggregates of fibers (Figure 2d–f; Figure S14a,b, Supporting Information). When the two samples were mixed, the resulting superstructured host–guest hydrogel exhibited 10–100 µm bundles throughout the gel's structure, similar to those observed by SEM, suggesting the addition of fluorophores did not significantly impact superstructure formation (Figure 2f; Figure S14c, Supporting Information). The emergence of the large bundles resulted in enhanced porosity throughout the depth of the gels imaged. The white and yellow color suggested the colocalization and possible reorganization of the Cy3‐labeled Ada‐E_2_ PA and Cy5‐labeled CD‐E_2_ PA within the superstructure bundles. Dylight 405‐labeled E_2_ PA was observed throughout the whole image indicating that the host–guest PAs are primarily responsible for the formation of the bundles. To further understand this self‐assembly driven bundling phenomenon, micrographs of the superstructured assembly were assessed for colocalization between the different dye‐labeled PAs utilizing the Pearson's correlation coefficient, which quantitatively compares pixel colocalization.^[^ ^47^ ^]^ Briefly, the Pearson's correlation coefficient measures the pixel‐by‐pixel covariance in the signal levels of two images; a perfect positive correlation is 1, no observed correlation is 0, and a perfectly inverse correlation is ‐1.^[^ ^48^ ^]^ By comparing the Pearson's correlation coefficients of the DyLight 405‐labeled E_2_ PA, Cy3‐labeled Ada‐E_2_ PA, and the Cy5‐labeled CD‐E_2_ PA in images of the superstructured material, an enhanced Pearson's correlation coefficient was evident for the Ada‐E_2_ PA with the CD‐E_2_ PA when compared to either of these with the E_2_ PA (Figure 2g). It is suggested that the increase in colocalization of the Ada‐E_2_ PA and the CD‐E_2_ PA is a result of reorganization via dynamic exchange of these molecules and thus their enrichment within the bundles. The E_2_ PA does not colocalize as well, implying there are regions of higher density of E_2_ PA molecules that do not overlap with the cyclodextrin or adamantane PAs and the most likely cause of this would be a redistribution of molecules as proposed above.

Control experiments of single, fluorescently labelled, PA samples did not reveal any appreciable colocalization between channels (Figure S15, Supporting Information). Hence, this quantitative analysis of the confocal Z‐stacks of the superstructured PA material provides evidence to support superstructure formation as a result of the host–guest interaction between the PA nanoribbons. To test the reversibility of the superstructure formation, a solution of 1‐adamantaneacetic acid (free adamantane) was used to outcompete the Ada‐E_2_ PA and dissociate the host–guest interaction with the CD‐E_2_ PA which resulted in a visible decrease in opacity of the sample (Figure S16, Supporting Information). Furthermore, as free adamantane was added to the superstructured host–guest hydrogel, the storage modulus decreased as well. With 0.5 equivalents of free adamantane added relative to the CD‐E_2_ PA, the modulus was 490 ± 22 Pa and with 1 equivalent the modulus decreased further to 110 ± 13 Pa (Figure 2h). This modulus is similar to that measured for the Ada‐E_2_ PA and the CD‐E_2_ PA prior to mixing (Figure 2c). SAXS analysis after addition of adamantane (Figure 2i) demonstrated that the peak at approximately 24.7 nm for the superstructured host–guest hydrogel disappeared entirely and the Guinier slope returned to ‐1.3, resulting in a scattering distribution more representative of the individual CD‐E_2_ PA and Ada‐E_2_ PA solutions (as shown in Figure 1h). By confocal microscopy, as 1 equivalent of adamantane was added to the superstructured host–guest hydrogel, bundle formation decreased throughout the gel (see Figure 2j). After the addition of 2 equivalents of adamantane, large bundles could not be observed and only very small aggregates of filaments of approximately 1 µm in width were observed, suggesting the dissociation of the host–guest interactions and the reversal of the enhanced porosity that was driven by the formation of the superstructures formed within the gel (Figure S17, Supporting Information). In summary, interactions between the Ada‐E_2_ PA and CD‐E_2_ PA and dynamic exchange drove superstructure formation, which in turn created not only morphological changes in the gels but also rheological changes as well. Dynamic exchange among the molecules allowed the formation of supramolecular polymers that concentrate host–guest interactions within the regions containing the bundled fibers of the superstructure (Figure 2k). Furthermore, the reversibility of this process was demonstrated through the addition of soluble guest molecules to displace inter‐fiber crosslinking.

### 2.3 Cellular Response to the Incorporation of Bioactivity in the Host–Guest Biomaterial

**Figure 3:**
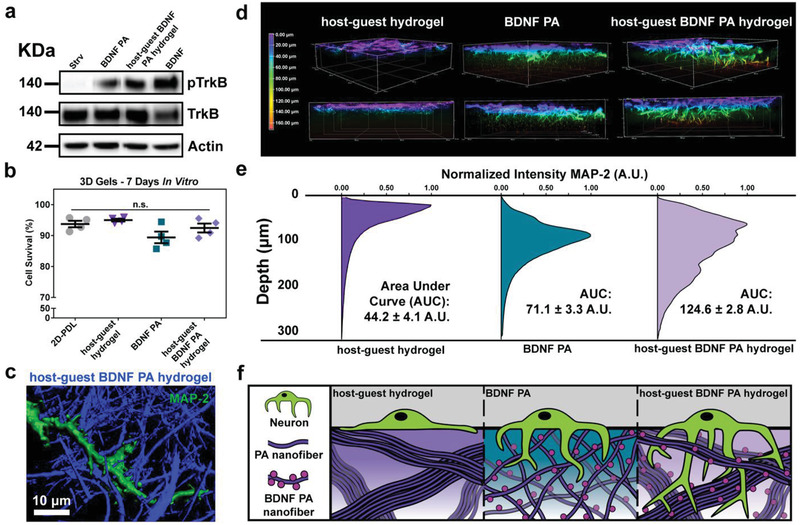
Effect of BDNF mimetic superstructured scaffolds on primary neuronal cultures. a) Western blot of phosphorylated TrkB (p‐TrkB), TrkB, and actin in neuronal cells treated in vitro with starvation media (Strv), BDNF PA, superstructured host–guest hydrogel containing BDNF mimetic signal (host–guest BDNF PA hydrogel), and BDNF protein (BDNF). b) Cell survival of neurons seeded on 2D poly‐d‐lysine coated coverslips, host–guest hydrogel, the BDNF PA, and the host–guest BDNF PA hydrogel after 7 days in vitro (values normalized to total number of cells). c) Imaris shadow projection of a neuron (MAP‐2, green) growing through superstructure bundles (blue). d) Depth‐coded z‐stack reconstructions showing cell infiltration on the host–guest hydrogel, the BDNF PA, and the host–guest BDNF PA hydrogel after 7 days in vitro. e) Pixel depth analysis and normalized average intensity of MAP‐2 under conditions described in (d); the area under the curve (AUC) is given with a standard deviation in arbitrary units (A.U.) for each hydrogel type. f) Schematic representation of cell infiltration in the various hydrogels (*p < 0.05, and **p < 0.01, LSD test (b) (*n* = 4), (e) (*n* = 3)). The BDNF PA was coassembled with the CD‐E_2_ PA at 10 mol% (10 mol% BDNF PA, 10 mol% cyclodextrin PA, and 80 mol% E_2_ PA) and separately with the Ada‐E_2_ PA (10 mol% BDNF PA, 10 mol% adamantane PA, 80% E_2_ PA). The two PAs were mixed in a 1:1 ratio to form a superstructure that incorporates the BDNF PA.

We evaluated the effect of the superstructure on bioactivity by incorporating a PA molecule in the supramolecular system that has been shown to mimic brain‐derived neurotrophic factor (BDNF PA).^[^ ^30^ ^]^ Our objective was to explore whether the addition of the host–guest moieties and their ability to create superstructures would interfere or enhance bioactivity. Thus the BDNF PA was coassembled with the CD‐E_2_ PA at 10 mol% (10 mol% BDNF PA, 10 mol% cyclodextrin PA, and 80 mol% E_2_ PA) and separately with the Ada‐E_2_ PA (10 mol% BDNF PA, 10 mol% adamantane PA, 80% E_2_ PA) (Figure S18a, Supporting Information). The BDNF PA was coassembled at 10 mol% within the host–guest hydrogel as this concentration had been shown previously to form thin nanoribbons and induce significant neuronal activity.^[^ ^30^ ^]^ The two PAs were mixed in a 1:1 ratio to form a superstructure that incorporates the BDNF PA. The properties of this supramolecular system do not differ significantly from the properties of the superstructure mixture without the BDNF PA (Figure S18b–g, Supporting Information). Because the BDNF PA was previously shown to activate the BDNF specific TrkB receptor,^[^ ^30^ ^]^ we also investigated the receptor phosphorylation using our supramolecular biomaterial. The host–guest BDNF PA hydrogel, BDNF PA, and BDNF protein alone were added to media to treat embryonic primary mouse cortical neurons in vitro, and the amount of phosphorylated receptor was quantified using Western blot analysis (**Figure** **3**a). We observed that the BDNF PA and BDNF superstructured materials induced similar levels of p‐TrkB and therefore conclude that its bioactivity is not compromised relative to the original BDNF PA (Figure S19, Supporting Information). To exclude the possibility of any cytotoxic effects of the CD‐E_2_ PA or Ada‐E_2_ PA, a cell viability assay was performed on primary cortical neurons seeded on 3D host–guest hydrogel scaffolds with and without the BDNF PA for 7 days in vitro. The cell survival remained above 80% for all conditions, which is indicative of a healthy primary culture (Figure 3b; Figure S20, Supporting Information).^[^ ^49^ ^]^

Next we investigated whether neurons could infiltrate gels of the BDNF superstructured material. We have previously reported the ability of the BDNF PA to increase infiltration of primary cortical neurons on 3D PA gels,^[^ ^30^ ^]^ so we evaluated if the superstructured material had an effect on BDNF PA‐induced infiltration. Gels were prepared in a mold to create them with uniform size and degree of swelling after media addition for each gel condition tested and were subsequently seeded with neurons. Under the conditions used for in vitro testing (25 × 10^−3^ m Ca^2+^ was added to induce ionic crosslinking of the BDNF PA hydrogel as the modulus without calcium is not self‐supporting (Figure S10a, Supporting Information)), the bioactive host–guest BDNF PA hydrogel, the BDNF PA alone, and the host–guest hydrogel all had a G′ between 1.2 and 1.4 kPa which is within the range of mechanical properties of neural tissue (Figure S21a, Supporting Information).^[^ ^50^ ^]^ The cells were grown for 1 week in vitro and the depth of infiltration of the neurons was analyzed using microtubule associated protein 2 (MAP‐2), a dendritic marker for phenotypic maturity. A shadow projection revealed that neurons were able to extend their dendrites and interweave them through the network of bundles within the superstructured material (Figure 3c). Only cells seeded on the BDNF PA and the host–guest BDNF PA hydrogel induced significant infiltration over 50 µm (Figure 3d; Figure S21b,c, Supporting Information). The host–guest BDNF PA hydrogel facilitated the growth of the most widely distributed network of neurons throughout the depth of the gel. This was shown by comparing the area under the curve (AUC) of normalized MAP‐2 intensity versus depth in the host–guest BDNF PA hydrogel, the BDNF PA, and the host–guest hydrogel having AUC values of 124.6 ± 2.8, 71.1 ± 3.3., and 44.2 ± 4.4, respectively (Figure 3e). We hypothesize that this greater infiltration in the host–guest BDNF PA hydrogel is due to two factors. First, it is necessary for the cells to sense the BDNF mimetic signal on the supramolecular polymers to induce any infiltration, which is supported by the lower infiltration in the case of the host–guest hydrogel without the BDNF PA incorporated. The striking differences in cell response on the host–guest hydrogel and its BDNF‐functionalized equivalent, which have the same storage modulus and are therefore the same from a mechanobiology perspective, suggest that cells are initially responding to the BDNF signal and the stiffness of the scaffold is not playing a critical role. Second, we propose that the ability of cells to infiltrate the bioactive material is enhanced by the additional porosity of the superstructured scaffold. Confocal z‐stacks were taken of E_2_‐DyLight 405 labeled gels, and shadow projections were used to analyze the various samples. It was found that the host–guest hydrogel and the host–guest BDNF PA hydrogel contained large pores as indicated by a significantly lower ratio of material volume to total scaffold volume (Figure S22, Supporting Information). We conclude that the BDNF signal in conjunction with enhanced porosity generated by the superstructure formation enables neurons to penetrate deeper into the PA network (Figure 3f). Taken together, these results show that the self‐assembly of the hierarchical superstructure is able to enhance neuronal bioactivity of PAs in vitro.

### 2.4 3D Printing of the Superstructured PAs

**Figure 4:**
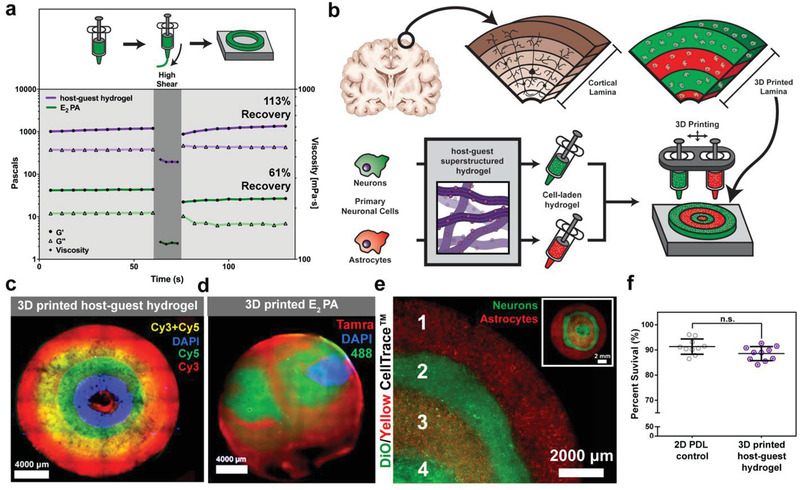
3D printed cortical brain‐like layered structures using superstructured inks. a) Storage modulus (G′), loss modulus (G″) and viscosity for the host–guest hydrogel and E_2_ PA before and after high shear deformation. b) Schematic representation of cortical lamina in the brain and a 3D printed version fabricated using cell‐laden host–guest hydrogel inks (top), and method of fabrication (bottom). c) Optical micrograph of a 3D printed pattern using the superstructured host–guest hydrogel and d) the E_2_ PA hydrogel labeled with different fluorophores in the following sequence from the outermost layer to the core: Cy3, Cy3 + Cy5, Cy5, DAPI, and Cy3. d) Optical micrograph of a 3D printed structure using the E_2_ PA hydrogel with concentric layers labeled with TAMRA‐E_2_ PA (red), Alexa 488‐E_2_ PA (green), and DAPI + E_2_ PA (blue). e) 3D printed pattern using the host–guest superstructured hydrogel (layers 1, 2, and 4) or its bioactive form with BDNF PA (layer 2) with Vybrant DiO labeled neurons (green), and Yellow Celltrace labeled astrocytes (red) or mixtures of both cells: 1) astrocytes, 2) neurons 3) astrocytes and neurons, and 4) neurons. f) Quantification of cell survival in a coculture of neurons and astrocytes on a 2D poly‐d‐lysine coated coverslip and within a 3D printed hydrogel scaffold containing the host–guest superstructured hydrogel (3 days in vitro, values normalized to total number of cells).

We investigated the effect of superstructure formation on the 3D printing capabilities of the peptide amphiphile gels. Our goal was to print the PA material into self‐standing objects without adding a layer of divalent cations to the printing surface^[^ ^51^ ^]^ which does not allow for gelation to occur beyond the first printed layer. We also wanted to avoid the need for incorporating additive molecules, as these may compromise some of the materials properties and could limit applications. The second objective was to utilize the ability of the superstructured material to be coassembled with bioactive epitopes in more complex forms for in vitro cell assays. 3D printing of neuronal cells in spatially defined scaffolds that can simulate the complexity of neural tissue for a variety of in vitro assays is an important but challenging goal.^[^ ^52^, ^53^ ^]^ For example, extruding neuronal cells is particularly challenging since the bioink carrier must be of appropriate softness to avoid shearing cells during extrusion while subsequently maintaining an appropriate stiffness to hold‐shape post‐extrusion.^[^ ^54^ ^]^ Creating a material that addresses this problem while presenting biologically relevant signals will create a versatile new platform for in vitro studies or tissue regeneration. Initially, we studied the thixotropy of the superstructured material and the E_2_ PA to determine their printability. We did this by conducting a rheological interval study to mimic the shear strain expected before, during, and after extrusion through a print head (**Figure** **4**a). During each phase, the storage and loss modulus were recorded for each material. After the crosslinks of the superstructured hydrogel are disrupted during high shear, its mechanical properties quickly recover to original values suggesting that the material's integrity could be maintained throughout the printing process, with a 113% recovery (before: *G*′ = 1199.8 Pa; after: *G*′ = 1361.5 Pa). Conversely, the E_2_ PA had a much lower storage modulus initially which then further decreased significantly after exposure to high shear forces, and only 61% recovery (before: *G*′ = 43.9 Pa; after: *G*′ = 26.8 Pa). The observed recovery of mechanical properties after high shear implies that the superstructured material is capable of retaining a 3D printed shape following extrusion.

Once we established the shear recovery properties of the superstructured hydrogel, we prepared multiple superstructured inks laden with different cell types and bioactive cues. These inks could be printed in concentric circles to mimic the natural organization of the layers found within the brain cortex as well as into ordered macroporous self‐supporting hydrogels (Figure 4b; Figure S23, Supporting Information).^[^ ^55^ ^]^ The addition of cells to the host–guest hydrogel did not visually change the properties of the material suggesting that the presence of cells did not impede host–guest interactions or restrict superstructure formation. To test if this was possible, several fluorophore‐labeled inks were used to allow visualization. When printed, the superstructured material held its shape and microscopy revealed clearly layers with distinct colors, confirming the possibility of printing complex patterns (Figure 4c; Figure S24a,b, Supporting Information). In contrast the 3D printed E_2_ PA behaved as a liquid droplet and the spatial resolution was highly dependent on the print speed (Figure S24c, Supporting Information) since the layers quickly deform as the liquid relaxed over time. Within the one hour elapsed between printing and imaging, the E_2_ PA samples did not contain distinct concentric layers (Figure 4d).

Next, primary cortical astrocytes and neurons — the two major cell types in the central nervous system (CNS) — were isolated from mice (at P0 and E16 respectively) and incorporated into the superstructured ink to further mimic the composition of CNS tissue, and tested for cell viability. Neurons were labeled with a membrane‐intercalating dye called Vybrant DiO and astrocytes with Yellow Celltrace prior to printing. Cells were printed in a sequential pattern to simulate the layer‐specific morphological and biochemical differences that prevail within the brain cortex as glial cells support neuron growth during development and beyond.^[^ ^56^ ^]^ The layers were also printed with the bioactive host–guest BDNF PA hydrogel and the different cell types. The 3D printed scaffolds were then cultured for 7 days in vitro and later imaged (Figure 4e). We found that after this period of time the cells were evenly distributed throughout the 3D printed layers and the host–guest hydrogels maintained spatial resolution of their layered pattern. Furthermore, during these experiments we did not find any evidence of hydrogel degradation, consistent with previous studies which have demonstrated the presence of peptide amphiphile hydrogels for two weeks in a relevant in vivo model of muscle regeneration.^[^ ^28^ ^]^ To assess cell viability, a live/dead assay was conducted 3 days after printing using calcein and propidium iodide, and the cell survival was quantified (Figure 4f; Figure S25, Supporting Information). The superstructured material had a cell survival of 89 ± 3% and was not statistically different from cells grown on the positive control which was a 2D poly‐d‐lysine (PDL) coated coverslip with a percent survival of 91 ± 3%, confirming that the 3D printing process did not substantially affect cell viability. This work demonstrated the ability of the superstructured materials to not only be printed into complex shapes, but also incorporate a bioactive signal and different cell types to better mimic tissue architectures.

## 3 Conclusions

We demonstrated the use of monomer exchange dynamics in mixtures of two supramolecular polymers to create a superstructured hydrogel containing domains with highly concentrated host–guest interactions. We found that these interactions can be molecularly tuned to obtain a specific storage modulus or to change it by adding molecules that disrupt the host–guest complex. The complexes can be disrupted under high shear to enable 3D printing of these materials but following recovery of the structure, printed objects are able to maintain their shapes and layered patterns. Finally, we investigated here the potential use of these hydrogels as bioactive supramolecular biomaterials by coassembling bioactive monomers that promote the infiltration of neurons and also activate a neuronal receptor.

## Conflict of Interest

The authors declare no conflict of interest.

## Author Contributions

A.N.E. and T.D.C. contributed equally to this work. T.D.C. and A.N.E. synthesized materials, designed and performed experiments, analyzed data, and wrote the manuscript. S.M.C. contributed to the material design and synthesis, performed experiments, analyzed data, and gave intellectual input. J.J.W.R synthesized materials, carried out experiments, and analyzed data. E.P.B. performed association constant measurements. Z.A. helped carry out experiments and took part in discussions. J.F.E. performed SEM, contributed intellectually, and created figure schematics. K.S.W. provided 3D printing expertise and created printing files. S.I.S. wrote the manuscript and supervised the research. The manuscript was written through contributions of all authors. All authors have given approval to the final version of the manuscript.

## Supporting information

Supporting Information

## Data Availability

Research data are not shared.
