# Supplementary material for: Superstructured Biomaterials Formed by Exchange Dynamics and Host–Guest Interactions in Supramolecular Polymers
Source: Adv Sci (Weinh). 2021 Feb 22;8(8):2004042. doi: 10.1002/advs.202004042 (PMC8061421; doi:10.1002/advs.202004042)
Supplement: Supplementary file 1 — Supporting Information [file ADVS-8-2004042-s001.pdf]

© 2021 Wiley-VCH GmbH

ADVANCED SCIENCE
Open Access

Supporting Information

for *Adv. Sci.*, DOI: 10.1002/advs.202004042

Superstructured Biomaterials Formed by Exchange Dynamics and Host-Guest Interactions in Supramolecular Polymers

Alexandra N. Edelbrock,[1,2,†] *Tristan D. Clemons,*[2,3,†] *Stacey M. Chin,*[3]
Joshua J.W. Roan,[4] *Eric Bruckner,*[4] *Zaida Álvarez,*[2,5] *Jack Edelbrock,*[2,4,5]
Kristen S. Wek,[4] *Samuel I. Stupp*[1,2,3,4,5*]

Supporting Information

Superstructured Biomaterials Formed by Exchange Dynamics and Host-Guest Interactions in Supramolecular Polymers

Alexandra N. Edelbrock,[1,2,†] Tristan D. Clemons,[2,3,†] Stacey M. Chin,[3] Joshua J.W. Roan,[4] Eric Bruckner,[4] Zaida Álvarez,[2,5] Jack Edelbrock,[2,4,5] Kristen S. Wek,[4] Samuel I. Stupp[1,2,3,4,5]*

Affiliations:

[1]Department of Biomedical Engineering, Northwestern University, Evanston, Illinois 60208, USA;

[2]Simpson Querrey Institute, Northwestern University, Chicago, Illinois 60611, USA;

[3]Department of Chemistry, Northwestern University, Evanston, Illinois 60208, USA;

[4]Department of Materials Science and Engineering, Northwestern University, Evanston, Illinois 60208, USA

[5]Department of Medicine, Northwestern University, Chicago, IL 60611, USA.

† *Authors contributed equally*

*Corresponding author. Email: s-stupp@northwestern.edu

Table of Contents

1. Synthesis **4**

1.1. Synthesis of 6-(6-aminohexyl)amino-6-deoxy-β-cyclodextrin (CD-HDA). 4

1.2. Synthesis of peptide amphiphiles. 6

1.2.1. General PA synthesis. 6
1.2.2. E_2 PA (E_2 PA). 7
1.2.3. Dylight 405 labeled E_2 PA. 8
1.2.4. TAMRA labeled E_2 PA. 8
1.2.5. Alexa Fluor® 488 labeled E_2 PA. 9
1.2.6. Adamantane PA (Ada PA). 9
1.2.7. Cy3 labeled Ada PA. 10
1.2.8. Cyclodextrin PA (CD PA). 11
1.2.9. Cy5 labeled CD PA. 12

2. Materials and Methods ***13***

2.1. Material Preparation. 13

2.1.1. Peptide Amphiphile Preparation. 13
2.1.2. Peptide Amphiphile 3D Gel Preparation. 14
2.1.3. Preparation of Free Adamantane Solution. 14
2.1.4. Preparation of 3D Printing Inks. 14

2.2. Material Characterization. 15

2.2.1. Cryogenic-Transmission Electron Microscopy (Cryo-TEM). 15
2.2.2. Fiber Width Analysis. 15
2.2.3. Dynamic Light Scattering (DLS). 16
2.2.4. Circular Dichroism (CD). 16
2.2.5 Fourier-transform infrared (FTIR) Spectroscopy 16
2.2.6. Determination of Association Constants by NMR Titrations. 17
2.2.7. Small Angle X-Ray Scattering (SAXS)/ Multi-Angle X-Ray Scattering (MAXS)/ Wide Angle X-Ray Scattering (WAXS). 18
2.2.8. Scanning Electron Microscopy (SEM). 19
2.2.9. Rheological Measurements. 19
2.2.10. Confocal Imaging of Peptide Amphiphile Materials. 20
2.2.11. Co-localization Analysis. 21

2.3. *In vitro* Studies. 21

2.3.1. Dissection of Embryonic Primary Cortical Neurons. 21
2.3.2. Dissection of Primary Astrocytes. 22
2.3.3. PA Treatments and Cell culture Procedures. 22

2.4. Biological Assays. 23

2.4.1. Western Blot. 23
2.4.2. Immunofluorescence. 24
2.4.3. Imaging. 24
2.4.4. Cell Viability. 25
2.4.5. Infiltration Study and Analysis of PA Gels. 25
2.4.7. Live Cell Dye Incorporation. 26
2.4.8. 3D Printing. 26
2.4.9. Statistical Analysis. 27

3. Supporting Figures ***28***

Figure S1. Adamantane and Cyclodextrin PA 100 mol% Analysis. 28
Figure S2. Fiber formation at differing PA co-assembly ratios by cryo-TEM. 29
Figure S3. Fiber formation at differing PA co-assembly ratios by conventional TEM. 29

Figure S4. Measurement of nanofiber diameters 30
Figure S5. X-ray scattering of 100 mol% peptide amphiphiles. 30
Figure S6. Fourier-transform infrared (FTIR) spectroscopy of peptide amphiphiles. 31
Figure S7. Determination of CD-E_2 PA and 1-adamantaneacetic acid binding constant. 32
Figure S8. Determination of Ada-E2 PA and β-cyclodextrin binding constant. 33
Figure S9. Determination of β-cyclodextrin and 1-adamantaneacetic acid binding constant. 34
Figure S10. Comparisons of storage moduli for different hydrogel conditions 35
Figure S11. Rheological Recovery Test of the superstructured mixture. 35
Figure S12. Fiber analysis of V_4-modified host-guest PAs. 36
Figure S13. Macroscopic characterization of V_4-modified host-guest PAs. 37
Figure S14. Split channels of the Ada-E_2 PA, CD-E_2 PA and superstructured mixture. 38
Figure S15. Pearson's correlation analysis of single fluorescent labelled control samples. 39
Figure S16. Photographs of superstructured host-guest hydrogel disassembly with free adamantane. 40
Figure S17. Fluorescent images of the superstructured host-guest hydrogel + 1 equivalent free adamantane and 2 equivalents free adamantane. 40
Figure S18. Characterization of the host-guest PA system with the incorporation of the BDNF PA. 41
Figure S19. TrkB receptor activation of primary cortical neurons treated with BDNF superstructure. 42
Figure S20. Live-Dead Analysis of embryonic primary cortical neurons seeded on 3D gels. 43
Figure S21. Rheological properties of PA scaffolds and infiltration of cortical neurons in PA scaffolds. 44
Figure S22. Shadow projection volume analysis of PA scaffolds. 45
Figure S23. Host-guest hydrogel macro-porous scaffold. 46
Figure S24. 3D printed concentric circles of the host-guest hydrogel. 47
Figure S25. Live-Dead assay images. 47

4. Spectroscopic Data ***48***

4.1. ^{1}H NMR spectrum of 6-*O*-monotosyl-6-deoxy-β-cyclodextrin (CD-tos). **48**

4.2. ^{1}H NMR spectrum of 6-(6-aminohexyl)amino-6-deoxy-β-cyclodextrin (CD-HDA). **48**

4.3. LCMS of the adamantane PA. **49**

4.4. LCMS of the cyclodextrin PA. **49**

4.5. LCMS of the BDNF PA. **50**

5. References ***51***

1. Synthesis

1.1. Synthesis of 6-(6-aminohexyl)amino-6-deoxy-β-cyclodextrin (CD-HDA).

CD-HDA was synthesized according to methods described previously by Loebel *et al.*[1] with a few minor modifications described below. β-Cyclodextrin (CD) (50 g, 44 mmol, 1 equiv) was suspended in milli-Q water (300 mL) and cooled to 0˚C in an ice bath. *p*-Toluenesulfonyl chloride (10.07 g, 53 mmol, 1.2 equiv) was dissolved in acetonitrile (25 mL). This solution was added dropwise by glass pipette to the CD suspension over 10 minutes under stirring. The reaction mixture was maintained at 0˚C for 2 hours under vigorous stirring. NaOH (5.3 g, 133 mmol, 3 equiv) was dissolved in milli-Q water (20 mL). The CD solution was removed from ice, and the NaOH solution was added dropwise by glass pipette to the CD solution under stirring. The CD solution was left at room temperature for a further 30 minutes under vigorous stirring. The pH was then adjusted by addition of solid ammonium chloride until the pH stabilized between 8.5-9 by pH paper. The solution was decanted into 50 mL conical tubes and cooled on ice. The solid was collected by centrifugation (3200 g, 2 min) and washed with water (2 × 40 mL), acetone (2 × 40 mL) and finally diethyl ether (1 × 40 mL) to afford the monotosylated product: 6-*O*-monotosyl-6-deoxy-β-cyclodextrin (CD-tos) (12.4 g, 25%). ^{1}H NMR (500 MHz, DMSO-d_6) δ = 2.43 (s, 3H), 3.0–3.75 (m, overlap with HOD), 4.10–4.72 (m, 6H), 4.77 (s, 2H), 4.83 (m, 7H), 5.51–5.93 (br s, 14H), 7.43 (d, 2H), 7.75 (d, 2H) ppm (see section 4.1. for NMR spectra). **MS** (ESI): m/z: calcd. for $[C_{49}H_{76}O_{37}S]^+$: $[M + NH_4]^+$ = 1307.20; found: 1306.6 $[M + NH_4]^+$

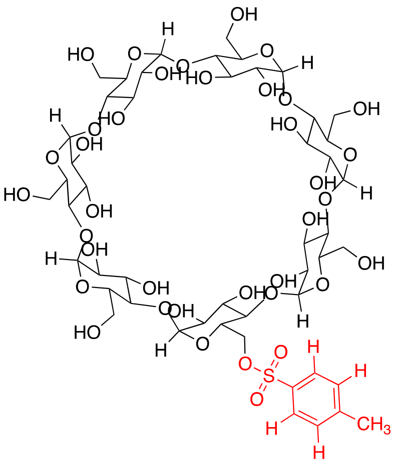

Structure of the 6-*O*-monotosyl-6-deoxy-β-cyclodextrin (CD-tos) intermediate

CD-tos (5.0 g, 3.9 mmol, 1 equiv) was dissolved in *N*,*N*-dimethylformamide (DMF) (25 mL) and placed under N_2 atmosphere. 1,6-hexanediamine (HDA) (17 mL, 172 mmol, 44 equiv) was heated to 60˚C and added via syringe to the CD-tos solution. The substitution reaction was carried out at 80˚C for approximately 20 hours. 5 mL of the reaction mixture was added to 50 mL conical tubes. Product was precipitated in ice cold acetone (40 mL). The product was centrifuged (3200 g, 2 min) and the supernatant decanted. The pellet was then re-dissolved in DMF (5 mL) and re-precipitated in cold acetone (40 mL). This process was repeated three times to ensure complete removal of unreacted HDA. The product was then washed in cold acetone (2 × 40 mL) followed by diethyl ether (2 × 40 mL) and dried under vacuum to afford 6-(6-aminohexyl)amino-6-deoxy-β -cyclodextrin (CD-HDA). ^{1}H NMR (500 MHz, DMSO-d_6) δ = 1.20–1.54 (m, 12H), 3.25–3.44 (m, overlaps with HOD), 3.51–3.75 (m, 28H), 4.44 (s, 6H), 4.83 (s, 7H), 6.64–5.81 (m,14H) ppm (see section 4.2. for NMR spectra). **MS** (ESI): m/z: calcd. for $[C_{48}H_{84}N_2O_{34}]^+$: $[M + 2H]^+$ = 617.59; found: 617.31 $[M + 2H]^+$

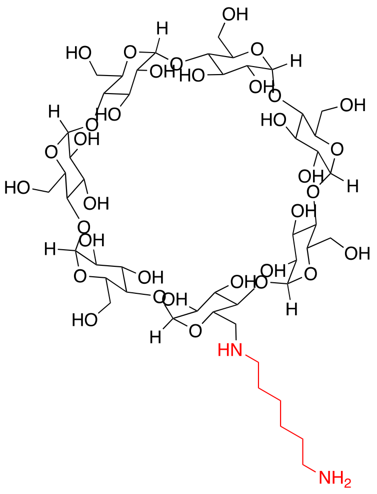

Chemical structure of the 6-(6-aminohexyl)amino-6-deoxy-β-cyclodextrin (CD-HDA) product

1.2. Synthesis of peptide amphiphiles.

1.2.1. General PA synthesis.

Peptide amphiphiles (PAs) were synthesized using standard Fmoc-solid-phase peptide chemistry. PAs were synthesized on Rink amide MBHA resin (EMD) with the majority of amino acid couplings performed in a CEM Liberty Blue microwave-assisted peptide synthesizer (CEM, Matthews, NC, USA). Fmoc groups were cleaved using 20% 4-methylpiperidine and 0.1 M hydroxybenzotriazole (HOBt) in *N*,*N*-dimethylformamide (DMF) at 90 °C for 30 s. Amino acids were coupled using 4 molar equivalents (equiv) of protected amino acid, 8 equiv ethyl cyanohydroxyiminoacetate (Oxyma) and 4 equiv of *N*,*N*'-diisopropylcarbodiimide (DIC) and 10 equiv *N*,*N*-diisopropylethylamine (DIEA) for 2–4 min at 90 °C in 50:50 DMF:DCM as solvent. Using this same procedure, palmitic acid (C_{16}) was conjugated to the N-terminus of the peptide as the hydrophobic tail.

Completed PA molecules were cleaved off the resin using a solution of 95:2.5:2.5 trifluoroacetic acid (TFA)/triisopropylsilane (TIPS)/water for 2–3 h. Volatile solvents were removed with rotary evaporation, and the PAs were precipitated with cold diethyl ether and dried

using a fritted filter. The PAs were then purified by preparative scale reverse phase high performance liquid chromatography (Shimadzu Prominence or Waters Prep 150), using a Phenomenex Gemini column (C-18 stationary phase, 5 µm, 100 Å pore size, either 30 × 150 mm or 50 × 250 mm). A mobile phase of acetonitrile and water was used, both containing 0.1% NH_4OH. Pure fractions were identified using electrospray ionization mass spectroscopy (ESI-MS) in positive or negative mode on an Agilent model 6520 Quadrupole Time-of-Flight (Q-ToF) using direct injection. MassHunter Workstation Data Acquisition software was used for instrument operation and MassHunter Qualitative Analysis software for data analysis and processing. Excess acetonitrile was removed with rotary evaporation, the samples were freeze-dried, and the powders were stored at -20°C until use.

The purity of PA molecules was confirmed using liquid chromatograph-mass spectroscopy (LC-MS), which was performed using an Agilent 1200 system with a Phenomenex Gemini C-18 column (100 × 1.00 mm; 5 µm) for basic conditions. The mass detector (MS) was an Agilent 6520 Q-TOF MS. All gradient methods followed: acetonitrile at 5% for 5 min at 50 µL/min, 5–95% over 25 min at 50 µL/min followed by 95% for 5 min at 50 µL/min. Ammonium hydroxide (0.1% v/v) for basic conditions was added to all solvents. Peaks were detected at λ = 220 nm.

1.2.2. E_2 PA (E_2 PA).

The following sequence $C_{16}V_2A_2E_2$ was synthesized on Rink amide MBHA resin making use of the CEM Liberty microwave-assisted peptide synthesizer and protocols described above in section 1.21.

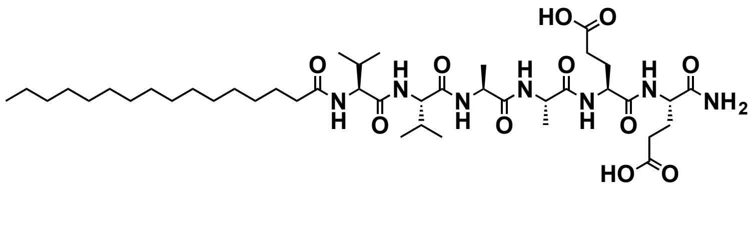

Chemical structure of the E_2 PA

1.2.3. Dylight 405 labeled E_2 PA.

The following sequence $C_{16}V_2A_2E_2C$ was synthesized on Rink amide MBHA resin making use of the CEM Liberty microwave-assisted peptide synthesizer and protocols described above in section 1.2.1. The purified PA was dissolved in tris(2-carboxyethyl)phosphine (TCEP) hydrochloride (5 equiv with respect to the PA) in pH 8 Tris buffer and reacted with maleimide-functionalized Dylight 405. The final product was purified by HPLC and stored until use as described in section 1.2.1.

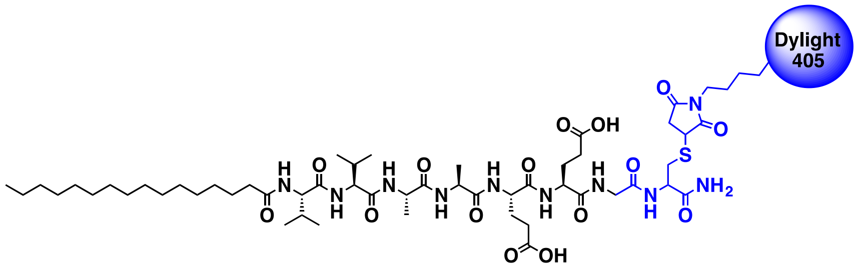

Chemical structure of the Dylight 405 labeled E_2 PA.

1.2.4. TAMRA labeled E_2 PA.

The following sequence $C_{16}V_2A_2E_2K$(Mtt) was synthesized on Rink amide MBHA resin making use of the CEM Liberty microwave-assisted peptide synthesizer and protocols described above in section 1.2.1. The terminal lysine ε-amine, protected with 4-methyltrityl (Mtt), was selectively deprotected on resin through the addition of a deprotection cocktail 3:5:92 TFA/TIPS/DCM, for multiple 5 min washes until yellow color was no longer seen in solution. Successful deprotection and subsequent coupling was verified through ninhydrin colorimetric assay (Kaiser test). Carboxytetramethyl rhodamine (TAMRA) (2 equiv) was coupled to the free ε-amine with 2 equiv TAMRA, 2 equiv PyBOP and 6 equiv DIEA in DMF for 16 h on a mechanical peptide shaker. Following successful coupling, the PA was cleaved, purified and stored as described in section 1.2.1.

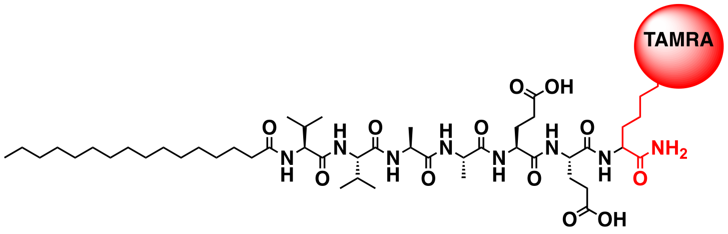

Chemical structure of the TAMRA labeled E_2 PA.

1.2.5. Alexa Fluor® 488 labeled E_2 PA.

The following sequence $C_{16}V_2A_2E_2C$ was synthesized on Rink amide MBHA resin using the CEM Liberty microwave-assisted peptide synthesizer and protocols described above in section 1.21. The purified PA was dissolved in tris(2-carboxyethyl)phosphine (TCEP) hydrochloride (5 equiv with respect to the PA) in pH 8 Tris buffer and reacted with maleimide functionalized Alexa Fluor® 488. The final product was purified by HPLC and stored until use as described in section 1.2.1.

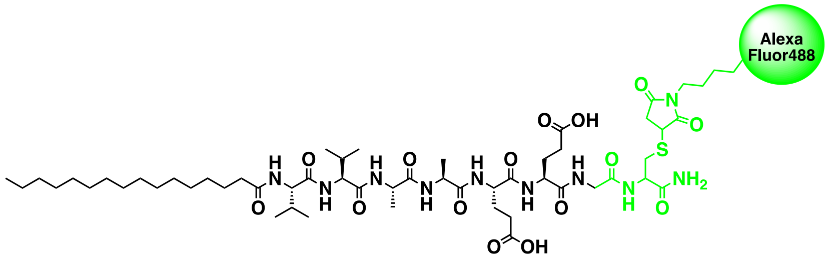

Chemical structure of the Alexa Fluor® 488 labeled E_2 PA.

1.2.6. Adamantane PA (Ada PA).

The following sequence $C_{16}V_2A_2E_4G_6K$(Mtt) was synthesized on Rink amide MBHA resin making use of the CEM Liberty microwave-assisted peptide synthesizer and protocols described above in section 1.2.1. The terminal lysine *ε*-amine, protected with 4-methyltrityl (Mtt), was selectively deprotected through the addition of a deprotection cocktail 3:5:92 TFA/TIPS/DCM, for multiple 5 min washes until yellow color was no longer seen in solution. Successful deprotection and subsequent coupling was verified through ninhydrin colorimetric assay (Kaiser test). 1-

Adamantanecarboxylic acid was coupled to the free ε-amine with 4 equiv 1-adamantanecarboxylic acid, 2 equiv PyBOP and 6 equiv DIEA in DMF for 4 h on a mechanical peptide shaker. Following successful coupling, the PA was cleaved, purified and stored as described in section 1.2.1.

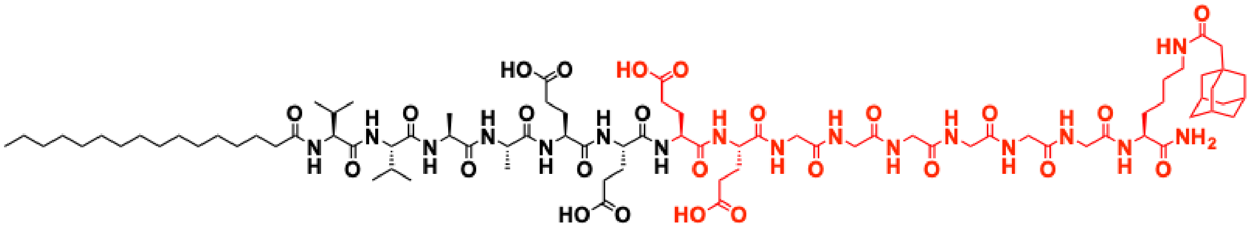

Chemical structure of the Adamantane PA (Ada PA).

1.2.7. Cy3 labeled Ada PA.

The following sequence $C_{16}V_2A_2E_4G_6K(Az)K(Mtt)$ (Mtt = 4-methyltrityl) was synthesized on Rink amide MBHA resin making use of the CEM Liberty microwave-assisted peptide synthesizer and protocols described above in section 1.2.1. The Mtt group on the terminal lysine ε-amine was selectively cleaved by the addition of a deprotection cocktail of 3:5:92 TFA/TIPS/DCM, for multiple 5 min washes until yellow color was no longer seen in solution. Successful deprotection and subsequent coupling was verified through ninhydrin colorimetric assay (Kaiser test). 1-Adamantanecarboxylic acid (4 equiv) was coupled to the free ε-amine with 2 equiv PyBOP and 6 equiv DIEA in DMF for 4 h on a mechanical peptide shaker. Following successful coupling, the PA was cleaved, purified and stored as described in section 1.2.1. DBCO-Cy3 was coupled to the purified PA through the azido lysine (K(Az)) (1.5 equiv) in DMF, at room temperature, protected from light overnight. Upon completion of the click reaction, the final product was purified by HPLC and stored until use as described in section 1.2.1.

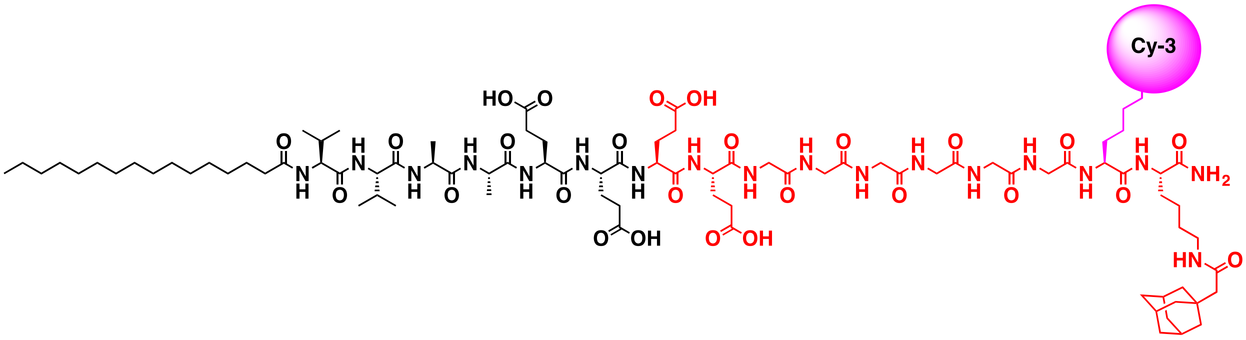

Chemical structure of the Cy3 labeled Ada PA.

1.2.8. Cyclodextrin PA (CD PA).

The following sequence $C_{16}V_2A_2E_4PEG_{10}K$(Mtt) was synthesized on Rink amide MBHA resin making use of the CEM Liberty microwave-assisted peptide synthesizer and protocols described above in section 1.2.1. The Mtt group on the terminal lysine *ε*-amine was selectively cleaved by the addition of a deprotection cocktail of 3:5:92 TFA:TIPS:DCM, for multiple 5 min washes until yellow color was no longer seen in solution. Successful deprotection and verified through ninhydrin colorimetric assay (Kaiser test). Diglycolic acid was coupled to the free *ε*-amine with 4 equiv diglycolic acid, 2 equiv PyBOP and 6 equiv DIEA in 50:50 DMF/DCM for 4 h on an automatic peptide shaker. Successful coupling was confirmed via by Kaiser Test. Following successful coupling, the resin was washed three times with DMF and 2 equiv PyBOP and 6 equiv DIEA in DMF was added to the resin for 5 min shaking. CD-HDA (synthesized as per protocol in section 1.1) was dissolved in DMF (2eq) and added to the activated PA solution and left to react on an automatic peptide shaker overnight. This coupling was repeated a second time to improve yield of the CD PA. Successful coupling was confirmed via ESI-MS before being cleaved, purified and stored as described in section 1.2.1.

MS (ESI): m/z: calcd. for $[C_{135}H_{235}N_{15}O_{67}]^+$: $[M + NH_4]^+$ = 3156.57; found: 3156.69 $[M + NH_4]^+$

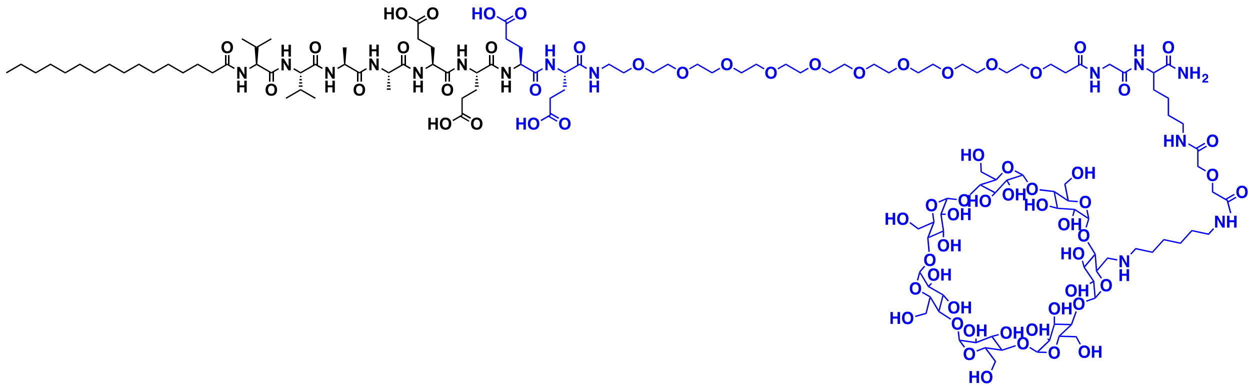

Chemical structure of the Cyclodextrin PA (CD PA).

1.2.9. Cy5 labeled CD PA.

The following sequence $C_{16}V_2A_2E_4PEG_{10}K(Az)K(Mtt)$ was synthesized on Rink amide MBHA resin making use of the CEM Liberty microwave-assisted peptide synthesizer and protocols described above in section 1.2.1. The Mtt group on the terminal lysine *ε*-amine was selectively cleaved by the addition of a deprotection cocktail of 3:5:92 TFA/TIPS/DCM, for multiple 5 min washes until yellow color was no longer seen in solution. Successful deprotection and subsequent coupling was verified through ninhydrin colorimetric assay (Kaiser test). Diglycolic acid was coupled to the free *ε*-amine with 4 equiv diglycolic acid, 2 equiv PyBOP and 6 equiv DIEA in 50:50 DMF/DCM for 4 h on an automatic peptide shaker. Following successful coupling, the resin was washed 3x with DMF and 2 equiv PyBOP and 6 equiv DIEA in DMF was added to the resin for 5 min shaking. HDA-CD (synthesized as per protocol in section 1.1) (2 equiv) was dissolved in DMF and added to the activated PA solution and left to react on an automatic peptide shaker overnight. This coupling was repeated a second time to improve yield of the CD PA before being cleaved, purified and stored as described in section 1.2.1. DBCO-Cy5 was coupled to the purified PA through the azido lysine (K(Az)) (1.5 equiv) in DMF, at room temperature, protected from light overnight. Upon completion of the click reaction, the final product was purified by HPLC and stored until use as described in section 1.2.1.

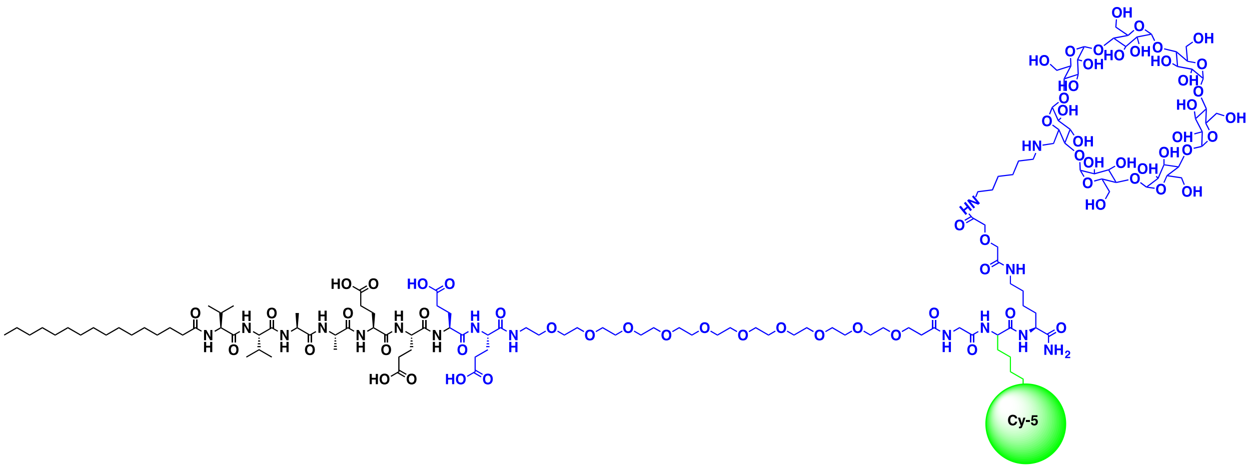

Chemical structure of the Cy5 labeled CD PA.

2. Materials and Methods

2.1. Material Preparation.

2.1.1. Peptide Amphiphile Preparation.

All PAs were taken from their powder form after lyophilization and dissolved in 125 mM NaCl and 3 mM KCl solution at a concentration of 1, 2, or 3 mg/100 µL. The resulting PA solution was then adjusted to a pH of 7.4 using 1 µL additions of 1 M NaOH. Once the pH was adjusted, Ada PA, CD PA, and the BDNF PA were co-assembled at 10 mol% concentrations with C_{16}-$V_2A_2E_2$ PA (E_2). The BDNF superstructure was made by co-assembling the CD PA or Ada PA at 10 mol% with the BDNF PA at 10 mol% and E_2 at 80 mol%. The CD-E_2 PA + BDNF PA and Ada-E_2 PA + BDNF PA were then mixed 1:1 for experiments. Similarly, the dye-labeled PAs were co-assembled with their respective non-labeled counterparts at 2 mol% of dye-labeled PA, 8 mol% CD-E_2 PA or Ada-E_2 PA, and 90% E_2 PA. After mixing, the solutions were heated with a polymerase chain reaction (PCR) thermocycler (Eppendorf) and annealed at 80 °C for 30 min, then slowly cooled at 1°C per minute back down to 27 °C.

2.1.2. Peptide Amphiphile 3D Gel Preparation.

Materials were prepared using annealed PAs previously described in section 2.1.1. To form superstructure gels, solutions of cyclodextrin and adamantane PAs were mixed together in the same Eppendorf using vigorous pipetting. Silicon isolators with adhesive (Invitrogen) were placed on coverslips coated with PDL and 100 µL of PA material was added to the circular well following previously published methods.[2] A porous membrane was placed on top of the well and a solution of 125 mM NaCl, 3 mM KCl and 25 mM $CaCl_2$ was added to each PA condition to ensure even swelling of all gels. Serum-containing media was then added to equilibrate the materials for cell-culture purposes.

2.1.3. Preparation of Free Adamantane Solution.

A 300 mM stock solution of 1-adamantaneacetic acid ("free adamantane") was prepared in dimethyl sulfoxide (DMSO). The stock solution was then diluted in 125 mM NaCl and 3 mM KCl solution to prepare a 1 mM and 2 mM free adamantane working solution used in rheological and confocal measurements. It was added in 0.5, 1, or 2 equivalents relative to the CD PA.

2.1.4. Preparation of 3D Printing Inks.

The E_2 PA print (Figure 4c) consisted of three different inks. The E_2 PA was co-assembled at 1 mol% with E_2 PA that had been functionalized with either Alexa 488 (Green) or TAMRA dye label (Red). The third ink consisted of E_2 mixed with a DAPI stain (Blue, 1:100 by volume dilution of DAPI into the material). For the superstructure layered print (Figure 4d), superstructure materials were prepared by mixing the CD PA and Ada PA at a 1:1 ratio and the different dye-labeled materials were incorporated at 1 mol% to obtain different layers. The four superstructure inks consisted of 1) the superstructure PA material with a Cy3 labeled Ada PA (Red), 2) a Cy5

labeled CD PA (Green), 3) both the Cy3 and Cy5 labeled materials (Yellow), and 4) a layer with DAPI stain mixed into the (Blue, 1:100 by volume dilution of DAPI into material).

2.2. Material Characterization.

2.2.1. Cryogenic-Transmission Electron Microscopy (Cryo-TEM).

300-mesh copper grids with lacey carbon film (Electron Microscopy Sciences, Hatfield, PA, USA) were glow discharged for 20 seconds in a PELCO easiGlow system (Ted Pella, Inc., Redding, CA, USA) prior to use. Samples at 1 w/v% were tenfold diluted to 0.1 w/v% immediately before blotting. 7 μL of sample solutions were transferred to the plasma-cleaned 300-mesh copper grids with lacey carbon support and plunge-frozen using a Vitrobot Mark IV (FEI) vitrification robot. Samples were blotted at room temperature (RT) with 95-100% humidity and plunge frozen into liquid ethane. Samples were transferred into a liquid nitrogen bath and placed into a Gatan 626 cryo-holder through a cryo-transfer stage. Cryo-TEM was performed using a liquid nitrogen cooled JEOL 1230 TEM working at 100 kV accelerating voltage. Images were acquired using a Gatan 831 CCD camera.

2.2.2. Fiber Width Analysis.

Using micrographs obtained from the Cryo-TEM imaging, the widths of at least 250 random fibers were measured by hand. Representative Cryo-TEM images used for the analyses are shown in Figure S2. Measurements were taken using the line tool in combination with the measuring feature in FIJI ImageJ analysis software.

2.2.3. Dynamic Light Scattering (DLS).

The Malvern Zetasizer Nano ZSP light scattering spectrometer was used for DLS measurements. Annealed PA samples were prepared as described in section 2.1.1. at 1 wt%. The sample was kept at a temperature of 25 °C and equilibrated for 30 seconds before each measurement which lasted 10 seconds each. The measurement angle was 173° backscatter and the attenuator and accumulation were automatically determined by the instrument for each run. Each measurement was repeated a total of three times.

2.2.4. Circular Dichroism (CD).

Samples were prepared as previously described in section 2.1.1. Each sample was diluted to concentrations between 0.01–0.04 wt% in milli-Q water. CD spectra were recorded on a JASCO model J-815 spectropolarimeter using a quartz cell of 0.5 mm optical path length. Continuous scanning mode was used with a scanning speed of 100 nm per minute with the sensitivity set to standard mode. High Tension (HT) voltage was recorded for each sample to ensure that the measurement was not saturated. An accumulation of three measurements was used and a buffer sample was background-subtracted to obtain final spectra. The final spectra were normalized to final concentration of each sample using a molar averaged molecular weight.

2.2.5 Fourier-transform infrared (FTIR) Spectroscopy

Samples were prepared as previously described in section 2.1.1, lyophilized and re-dissolved in D_2O to a final PA concentration of 10mM. The liquid samples were sandwiched between two CaF_2 windows using a spacer of 50 µm in an FTIR liquid flow cell (Pike Technologies). Infrared (IR) spectra were recorded on a Bruker model Tensor 37 spectrometer.

2.2.6. Determination of Association Constants by NMR Titrations.

The association constant for the binding between 1-adamantaneacetic acid and β-cyclodextrin was measured in 125 mM NaCl and 3 mM KCl buffer at pH 7.2–7.4. In a typical titration experiment, small amounts of a "guest solution" (100 mM 1-adamantaneacetic acid and 3.5 mM β-cyclodextrin in deuterated buffer) were added to approximately 0.8 ml of "host solution" (3.5 mM β-cyclodextrin in deuterated buffer) until at least 5 equiv of "guest" molecules were added. ^{1}H-NMR spectra were obtained at 25°C upon each addition of the "guest" solution and a binding isotherm was constructed by monitoring the protons on 1-adamantaneacetic acid.

The association constant for the binding between CD-E_2 PA and 1-adamantaneacetic acid was measured in 125 mM NaCl and 2 mM KCl buffer at pH 7.2-7.4. The "host solution" containing the CD-E_2 PA was prepared by mixing 1 wt% solutions of CD PA and E_2 PA in deuterated buffer such that the final solution would contain 80 mol% E_2 PA and 20 mol% CD PA (i.e. 1.4 mM CD-E_2 PA). The "host solution" was then annealed in a water bath at 80°C for 30 min, then slowly cooled to RT over 18 h. The "guest solution" was prepared by dissolving 1-adamantaneacetic acid in a portion of the "host solution" to obtain a solution containing 65 mM 1-adamantaneacetic acid and 1.4 mM CD-E_2 PA in deuterated buffer. In a typical titration experiment, small amounts of the "guest solution" were added to approximately 0.5 ml of "host solution" until at least 5 equiv of "guest" molecules were added. ^{1}H-NMR spectra were obtained at 25°C upon each addition of the "guest solution" and a binding isotherm was constructed by monitoring the protons on 1-adamantaneacetic acid.

The association constant for the binding between Ada-E_2 PA and β-cyclodextrin was measured in 125 mM NaCl and 2 mM KCl buffer at pH 7.2-7.4. The "host solution" containing the Ada-E_2 PA was prepared by mixing 1 wt% solutions of Ada PA and E_2 PA in deuterated buffer such that the final solution would contain 80 mol% E_2 PA and 20 mol% of Ada PA (i.e. 2 mM

Ada-E_2 PA). The "host solution" was then annealed in a water bath at 80°C for 30 min, then slowly cooled to RT over 18 h. The "guest solution" was prepared by dissolving 1-adamantaneacetic acid in a portion of the "host solution" to obtain a solution containing 35 mM β-cyclodextrin and 2 mM Ada-E_2 PA in deuterated buffer. In a typical titration experiment, small amounts of the "guest solution" were added to approximately 0.5 ml of "host solution" until at least 5 equiv of "guest" molecules were added. ^{1}H-NMR spectra were obtained at 25°C upon each addition of the "guest solution" and a binding isotherm was constructed by monitoring the protons on β-cyclodextrin.

The binding isotherms were fit to a standard 1:1 host-guest binding model (Eq. 1–3) where **[HG]** is the concentration of host-guest complex, **$[H]_0$** is the total concentration of "host" molecules, **$[G]_0$** is the total concentration of "guest" molecules, **δ_0** is the proton peak position at $[HG]/[H]_0 = 0$, **δ** is the proton peak position measured at different values of $[HG]/[H]_0$, and **δ_∞** is a fitting parameter. All spectra were obtained with a X500 Bruker Avance III HD system equipped with a TXO Prodigy probe and analyzed with the MestreNova (Mestrelab Research) software.

$$[HG] = \frac{1}{2}\left\{\left([G]_0+[H]_0+\frac{1}{Ka}\right)-\sqrt{\left([G]_0+[H]_0+\frac{1}{Ka}\right)^2-4[H]_0[G]_0}\right\} \quad 1$$

$$\delta = \delta_\infty\left(\frac{[HG]}{[H]_0}\right)+\delta_o \quad 2$$

$$\Delta\delta = \delta - \delta_o \quad 3$$

2.2.7. Small Angle X-Ray Scattering (SAXS)/ Multi-Angle X-Ray Scattering (MAXS)/ Wide Angle X-Ray Scattering (WAXS).

Experiments were performed at beamline 5-ID-D of the DuPont-Northwestern-Dow Collaborative Access Team (DND-CAT) Synchrotron Research Center at the Advanced Photon Source, Argonne National Laboratory. PA samples were prepared at 2 w/v% irradiated for 2 or 10 seconds. Data

was collected with an X-Ray energy at 17 keV (l = 0.83 Å). Sample to detector distances were as follows: 201.25 mm for SAXS, 1014.2 mm for MAXS, and 8508.4 mm for WAXS. The scattering intensity was recorded in the interval 0.002390 < q < 4.4578 $Å^{-1}$. The wave vector q is defined as = $(4\pi/\lambda)\ \sin(\theta/2)$, where θ is the scattering angle. Azimuthal integration (Fit2D) was used to average 2D scattering images to produce 1D profiles of intensity versus q. Samples were oscillated with a syringe pump during exposure to prevent beam damage. Background scattering patterns were obtained from samples containing 125 mM NaCl and 3 mM KCl. This background data was then subtracted from experimental data. All data was analyzed using the Irena software package running on IgorPro software.

2.2.8. Scanning Electron Microscopy (SEM).

All materials were gelled with 125 mM NaCl, 3 mM KCl and 25 mM $CaCl_2$ to ensure that all materials, even weaker controls, could be processed for SEM. These PA gels were fixed in a 2.5% glutaraldehyde (GTA) 4% paraformaldehyde (PFA) phosphate buffered saline (PBS) solution for 20 minutes. They were dehydrated in a series of ethanol solutions increasing in concentration from 30-100%. Ethanol was then removed with critical point drying using a Tousimis Samdri-795. Dehydrated samples were mounted on stubs using carbon glue and then coated with 16 nm of osmium (Filgen, OPC-60A) to create a conductive surface. Images were taken using a Hitachi SU8030 instrument using an accelerating voltage of 2 kV.

2.2.9. Rheological Measurements.

PA materials were prepared and annealed using methods described in section 2.1.1. An Anton Paar MCR302 Rheometer with a 25 mm cone plate was used for all rheological studies. 150 µL of PA liquid was placed on the sample stage. For experiments using Free Adamantane, 30 µL of either 1

mM or 2 mM adamantane solution (prepared as described in section 2.1.3) was added in droplets to the top plunger. The plunger was then lowered to the measuring position and a humidity collar was added during the “sample trim” phase to prevent sample evaporation during the measurement. The sample was equilibrated for 30 minutes with a constant angular frequency of 10 rad/s and 0.1% strain. The storage and loss modulus were recorded. For thixotropy studies, the sample was applied to the stage as previously mentioned. The first and third interval consisted of 10, 6 second points with an oscillating shear strain of 0.1% and angular frequency of 10 rad/s. The storage and loss modulus were plotted against time. The second interval consisted of 10, 1 second points with a constant shear rate of 100 s^{-1}. The viscosity was plotted against time. For recovery experiments, G’ and G’’ were measured during alternating intervals of low and high strain. The low strain interval was 200 seconds at 0.5% strain and the high strain interval was 50 seconds at 50% strain.

2.2.10. Confocal Imaging of Peptide Amphiphile Materials.

Dye-labeled PAs were prepared as previously described in section 2.1.1. 50 µL of CD-E_2 PA and Ada-E_2 PA labeled with fluorophores were mixed 1:1 directly before imaging. To image, a 10 µL droplet of each PA sample was placed on the bottom of a 35 mm glass petri dish (MatTek). Images were immediately taken using the Nikon A1R confocal microscope. Every ten minutes of imaging, a new droplet was placed to avoid drying effects. Z-stacks were made for each sample and processed using FIJI by ImageJ. For the unbundling experiments, 0.5 or 1 equivalent of free adamantane solution (prepared as described in section 2.1.3.) was added to the superstructure PA material and immediately imaged.

2.2.11. Co-localization Analysis.

Pearson's correlation coefficient analysis was performed on single slices of confocal Z-stacks collected on the dye labeled PAs. Analysis was performed in FiJi making use of the JaCOP plugin.[3] For this analysis, single focal plane slices from the confocal superstructure Z-stacks were isolated and the fluorescent channels from the corresponding PAs separated into individual images for pixel colocalization analysis. 10 separate Z-stack images of the superstructure PA assembly were analyzed, with 3 different focal planes taken from the central region of each stack. Results are displayed as means ± SD. Data was analyzed using the GraphPad Prism version 6.0 data management software to conduct ANOVA on groups of data. Statistically significant differences between each comparison were determined using Turkey's post hoc test (*$p<0.05$, **$p<0.01$, ***$p<0.001$).

2.3. *In vitro* Studies.

2.3.1. Dissection of Embryonic Primary Cortical Neurons.

All neurons used were obtained by dissecting embryonic brains following procedures described previously.[4] Timed-pregnant mice were anesthetized using isoflurane and sacrificed by cervical dislocation. The embryos were extracted at embryonic day 16 (E16). The cerebral cortices were extracted from the mouse embryos and the meninges were removed. The tissue was kept in a cold sterile solution of Hank's Balanced Salt Solution (HBSS) with 1% pen-strep (Invitrogen). The tissue was then digested using trypsin (Invitrogen) and DNAse I (Sigma-Aldrich) for 10 min at 37 °C. The cortices were broken up by pipetting up and down, centrifuged at 1000 g for 5 min, and resuspended in CO_2-equilibrated Neurobasal (NB, Invitrogen) neuronal culture medium with 10% normal horse serum (NHS, Invitrogen), 1% penicillin-streptomycin (pen-strep, Invitrogen), 0.5 mM L-glutamine (Invitrogen), and 5.8 µL/mL $NaHCO_3$ (Sigma-Aldrich). After centrifugation, the

cells pellet was resuspended and pre-plated at 37 °C for 30 min. The supernatant was collected and passed through a cell strainer with 100 µm pores. The resulting solution was centrifuged at 1000 g for 5 min. This pellet was resuspended in NB culture medium (1% NHS, 1% pen-strep, 0.5 mM L-glutamine, 22 µM glutamic acid (Sigma-Aldrich), 2% B27 (Gibco), and 5.8 µL/mL $NaHCO_3$ (Sigma-Aldrich)), and plated at different densities (depending on the type of experiment, see below). Tissue culture plastic or glass coverslips used in experiments with these cells were coated with poly-D-lysine (PDL, Sigma-Aldrich). After 24 h, the medium was replaced with serum-free neuronal culture medium (1% pen-strep, 0.5 mM L-glutamine, 2% B27, 5.8 µL/mL NaHCO3). These conditions allowed us to obtain a neuron-rich culture containing approximately 10% glial cells.

2.3.2. Dissection of Primary Astrocytes.

Glial cells were dissected from the cerebral cortex of newborn mice (postnatal day 0, P0), as described elsewhere.[5] In dissection buffer of HBSS with 1% pen-strep (Invitrogen), the meninges were removed, and the cortexes were separated out. This tissue was then digested using trypsin and DNAse I for 10 min at 37 °C. Dissociation of the tissue was carried out in Dulbecco's Modified Eagle Medium (DMEM, GIBCO), 10% NHS, 1% pen-strep, and 2 mM L-glutamine. Cells were centrifuged at 1000 g for 5 min, supernatant was removed, and cells were resuspended. Cells were then plated and grown to confluence at 37 C, 5% CO_2 for approximately 25-30 days *in vitro*. All experiments were performed using glial cells from the first through third passages (Ps1-Ps3). Cells were cultured at a density of 1 or 2 million cells per 100 µL of PA ink used for 3D printing.

2.3.3. PA Treatments and Cell culture Procedures.

Treatments were prepared by dissolving PA (BDNF PA, superstructure PA, or BDNF superstructure) in media without serum or B27 supplement. The total concentration of BDNF PA

in each condition was 10 µM and the total concentration of PA was 100 µM. Human/Murine/Rat BDNF protein (Peprotech) was resuspended at 0.25 nM in starvation media as a positive control. For Western blot experiments, primary cortical neurons were cultured in 6 well plates at a density of approximately 900,000 cells/well for 7 days *in vitro* (DIV) before being treated. Treatments were added for 24 h *in vitro* before protein was harvested. For infiltration and gel viability studies, 300,000 primary neurons per well were seeded on gels in a 24 well plate and cultured for 7 DIV. Samples were fixed in 4% PFA for 20 min at RT for immunofluorescence studies. For 3D printing experiments, primary astrocytes were seeded at 1 or 2 million cells/100 µL of PA. Primary neurons were seeded at 4 million cells/100 µL of PA. Prints were cultured for 7 or 14 DIV. Samples were fixed in 4% PFA for 20 min at RT.

2.4. Biological Assays.

2.4.1. Western Blot.

Halt Protease and Phosphatase Inhibitor Cocktail (Thermo Scientific) was used to extract protein from primary neuronal cultures. Samples were stored at -20 °C and a BCA assay (Thermo Scientific) was performed to determine protein content for each collected sample. Protein was then diluted with H_2O to ensure the same concentration for each condition. Samples were loaded into a 4-20% SDS-PAGE gel (Bio-Rad) and separated for 1.5 h using a power source set to 115 volts. The protein was electro-transferred from the SDS-PAGE gel to a nitrocellulose membrane (Bio-Rad). The membrane was checked for protein content using ponceau stain (Sigma-Aldrich). Once protein content was determined to be present, the membrane was blocked for 30 minutes using a 5% milk solution (Bio-Rad). The membrane was then incubated over night with primary antibodies. The following primary antibodies were used: rabbit anti-pTrkB (1:1000, Cell Signaling), rabbit anti-TrkB (1:1000, Cell Signaling), rabbit anti-Actin (1:2000, Sigma-Aldrich),

mouse anti-Actin (1:2000, Sigma-Aldrich). Corresponding secondary HRP-conjugated antibodies (1:1000, ThermoFisher) were incubated at RT for 2 h. Radiance Bioluminescent ECL substrate (Azure Biosystems) was used to detect protein signals. The membranes were imaged using the Azure Biosystems imager on the automatic setting. Densitometry analysis, was performed using ImageJ software and was standardized to total receptor or actin content as a control for protein loading.[6] Triplicate samples were analyzed from at least two separate experiments.

2.4.2. Immunofluorescence.

Fixed samples (4% PFA for 20 min at RT) were incubated with primary antibodies over night at 4 °C. Alexa 488 and Alexa 555 antibodies at a dilution of 1:2000 (Invitrogen) were incubated for 2 h at RT. The following primary antibodies were used: rabbit anti-MAP-2 (marker for mature neurons 1:2000, BioLegend). DAPI was used to stain the nuclei (nuclear stain, 1:2000, Invitrogen).

2.4.3. Imaging.

A Nikon A1R confocal laser-scanning microscope with GaAsP detectors was used to visualize and image fluorescent 3D samples. Resulting images were processed and converted to TIF files using a plugin through FIJI an ImageJ software (National Institutes of Health). Confocal z-stack reconstructions were compiled using NIS Elements Advanced Research Microscope Imaging software (version 4.20) or Imaris program (version 9.3.1, Bitplane Scientific software) for 3D interactive data viewing. Large fluorescent images of 3D printed PA gels were taken using a Nikon Ti2 Widefield Microscope. For visualization purposes, benchtop images of experiments visible to the human eye were taken using an Apple iPhone 6s. The images were placed in Adobe Photoshop (v.20.0.6) and adjustments for contrast, brightness and color balance were made to obtain optimal and consistent visual reproduction of the data.

2.4.4. Cell Viability.

Cell culture medium was removed, and the gels were rinsed once with HBSS. A calcein-AM/propidium iodide live/dead assay (Invitrogen) was used to check cell viability of cells seeded on top of or within 3D printed PA gel scaffolds. Calcein-AM (16 µM)/propidium iodide (20 µM) solution in HBSS was added to each well. The plate was covered with foil and left at RT for 15 min. The solution was removed, and the samples were rinsed with HBSS and the samples were immediately imaged using confocal microscopy.

2.4.5. Infiltration Study and Analysis of PA Gels.

PA materials were prepared as described in section 2.1.1. 3D Gels were prepared as described in section 2.1.2. to mimic the molded scaffold setup used *in vitro*. They were rinsed once with media and cells were seeded at 300,000 cells/well. Cells were incubated at 37 °C for one week *in vitro*. At the end of the study, cells were fixed with 4% PFA and stained with MAP-2 and DAPI using immunocytochemistry methods previously described in section 2.4.2. To image, gels were placed face-down on a glass bottom dish and images were taken using the Nikon A1R confocal microscope. 150 µm thick Z-stack projections of each gel were collected by confocal microscopy. Depth analysis was performed on the confocal Z-stacks using the ImageJ software, and the MultiMeasure function within the ROI manager to measure the depth of pixels. The area under the curve (AUC) was calculated using Graphpad Prism version 6.0 data management software and the standard deviation was reported in A.U.

In order to analyze the fiber architecture of the superstructure, BDNF PA, E_2 PA and BDNF superstructure, a 405-labeled E_2 PA was incorporated at 1 mol%. Again, materials and gels were prepared as described in sections 2.1.1 and 2.1.2. To image the samples, a Nikon A1R confocal

microscope with a 60x objective and a 2x Nyquist zoom was utilized for image capture. Each image was 15 µm in depth with a 0.25 µm step size. Images were then processed using Imaris shadow projection software. Using the statistics tab, the total volume of the field of view and the volume of the 405-labeled material within each z-stack projection was calculated.

2.4.7. Live Cell Dye Incorporation.

CellTrace^{TM} CFSE or CellTrace^{TM} Yellow dye were dissolved in DMSO at a concentration of 5 mM (ThermoFisher). After passaging, astrocytes were spun down and resuspended in PBS. The CellTrace dye was added at a working concentration of 5 µM and incubated at 1 million cells/mL 37 °C for 20 min. Concurrently, neurons were dissected following protocol described in section 2.3.1. and immediately resuspended in PBS at a concentration of 1 million cells/ml. Vybrant^{TM} DiO (ThermoFisher) was used to label neurons and 5 µL of dye was added per mL of cell suspension. Cells were incubated for 20 min at 37 °C. After incubation in their respective dyes, both types of cells were washed with PBS and media several times before resuspending them at the desired concentration within the superstructure PA and BDNF superstructure 3D printing inks.

2.4.8. 3D Printing.

The superstructure PA ink or BDNF superstructure ink was prepared by mixing the CD-E_2 PA and the Ada-E_2 PA solutions. Dyed cells were then mixed into the PA inks at the desired concentration and pipetted into the 3D printing syringes (Nordson EFD). 25 mm plastic coverslips (Thermanox) were placed inside 30 mm plastic petri dishes (Corning). Silicon isolators (Grace BIO-LABS) with a 20 mm diameter hole were used to hold the coverslip in place and guide the alignment of the 3D printer (BioX by CELLINK). A black dot was placed in the center of the silicon circle on the underside of the petri dish to help keep a consistent center point while printing. The main print

design consisted of concentric circles filling the 20 mm well in the silicon isolator. The PA, or PA-Cell inks were extruded through a 0.41 mm inner diameter nozzle (Nordson EFD). The printer temperature was set to 37 °C. A print speed of 3 mm/s and pressure of 10-20 kPa was used. Each ring consisted of 4 stacked layers of approximately 0.5 mm in height to create a total height of 2 mm. Gels containing cells were taken to a tissue culture hood and 4 mL of neuronal culture medium (previously described in section 2.3.4. was added to each dish. Gels were incubated for 7 DIV and then fixed with 4% PFA for 20 min at RT before being imaged on the Nikon Ti2 Widefield microscope or Nikon A1R confocal laser-scanning microscope.

2.4.9. Statistical Analysis.

Error bars shown indicate the standard error of the mean. Graphad Prism v.6 software was used to perform all statistical analysis. Analysis of variance (ANOVA) was used for all multiple group experiments with a Bonferroni post hoc test. P-values <0.05 were used to deem significance.

3. Supporting Figures

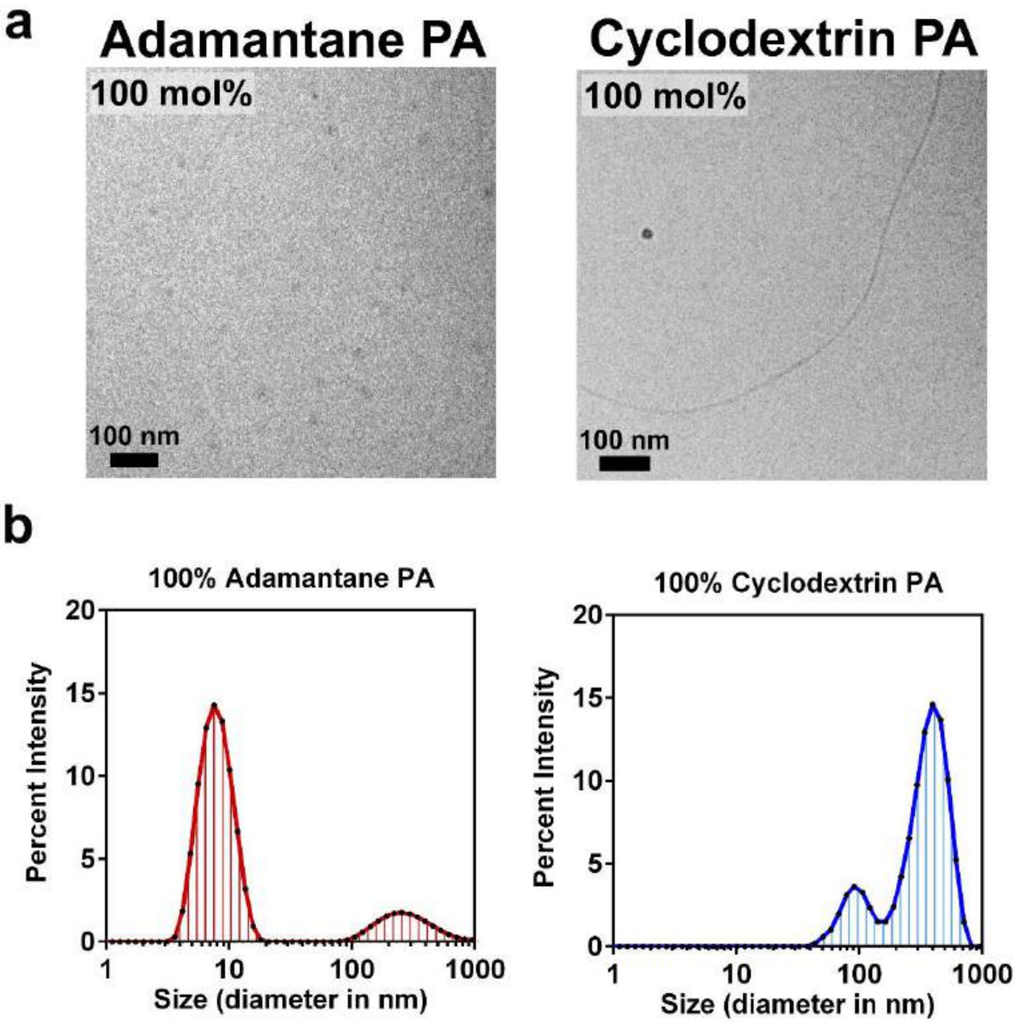

Figure S1. Adamantane and Cyclodextrin PA 100 mol% Analysis. (a) Cryo-TEM images of 100 mol% of the Adamantane PA (Ada PA, left) and the Cyclodextrin PA (CD PA, right). (b) Dynamic light scattering (DLS) spectra of 100 mol% Ada PA (left) and CD PA (right).

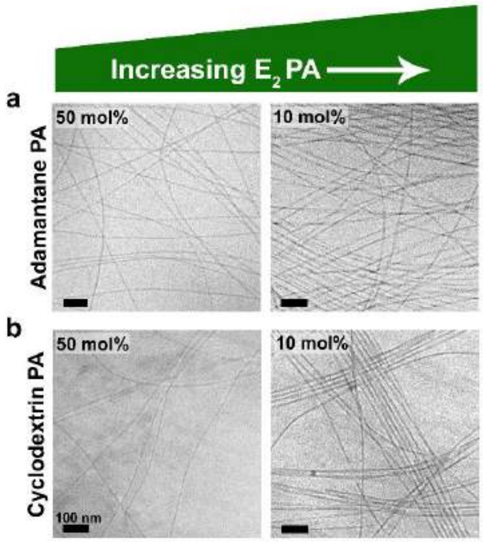

Figure S2. Fiber formation at differing PA co-assembly ratios by cryo-TEM. Cryo-TEM images of 50 mol% (left) and 10 mol% (right) of (a) the Adamantane PA (Ada PA) co-assembly and (b) the Cyclodextrin PA (CD PA) co-assembly with E_2 PA.

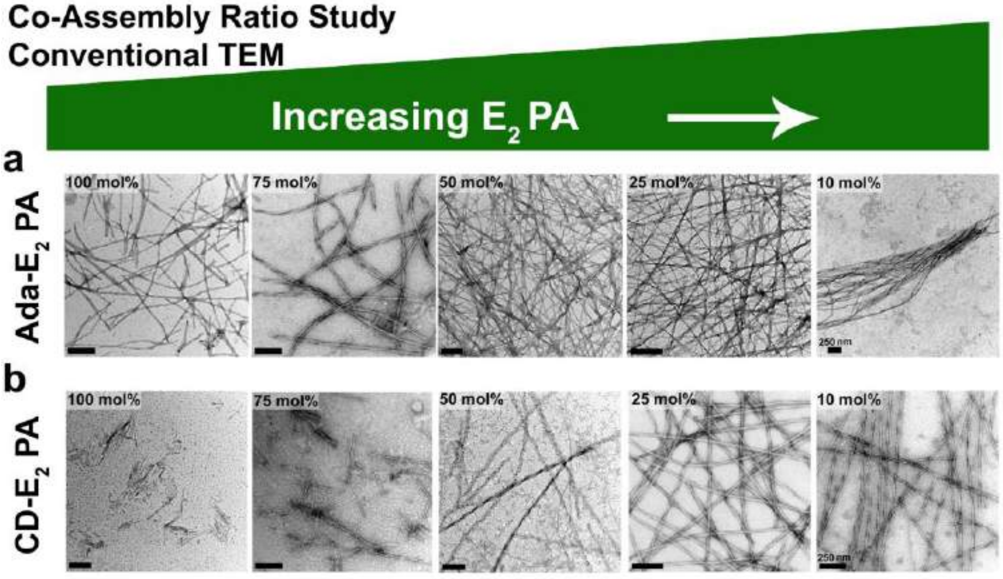

Figure S3. Fiber formation at differing PA co-assembly ratios by conventional TEM. TEM images of 100, 75, 50, 25 and 10 mol% of (a) Ada-PA with E_2 PA and (b) CD-PA with E_2 PA.

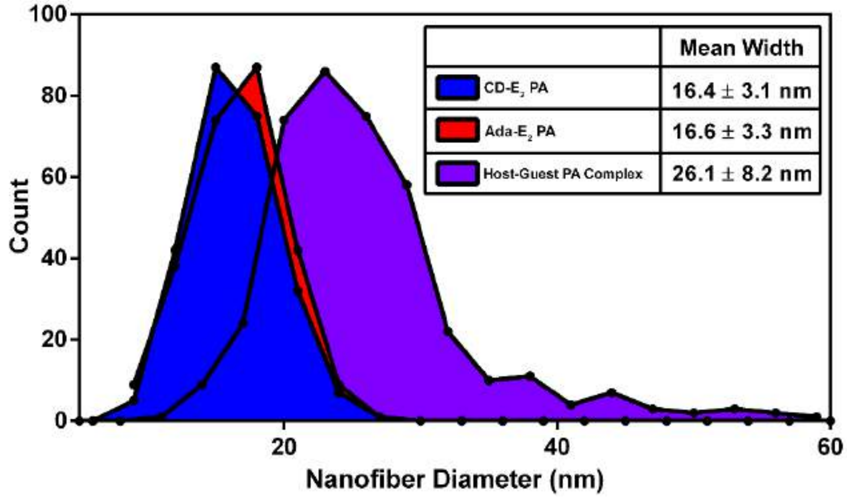

Figure S4. Measurement of nanofiber diameters. Measurement of nanofiber diameter including the mean and standard deviation, in nm, of the CD-E_2 PA, Ada-E_2 PA and Host-Guest PA Complex from representative Cryo-TEM images.

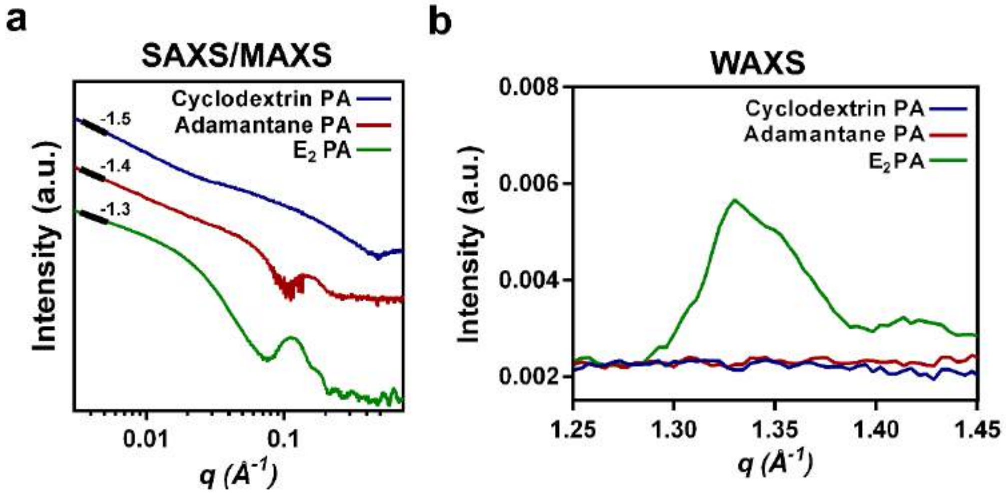

Figure S5. X-ray scattering of 100 mol% peptide amphiphiles. (a) Small angle x-ray scattering (SAXS)/mid angle x-ray scattering (MAXS) and (b) Wide angle x-ray scattering (WAXS) pattern of 100 mol% cyclodextrin PA (CD PA, Blue), 100 mol% adamantane PA (Ada PA, Red) and 100 mol% E_2 PA (Green).

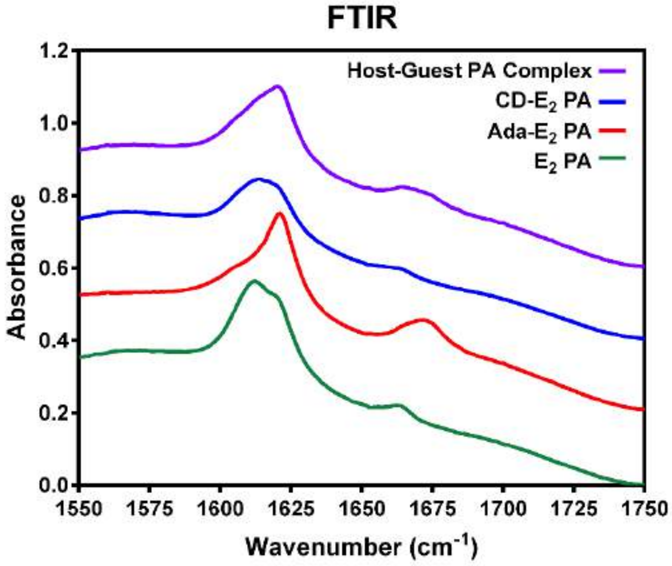

Figure S6. Fourier-transform infrared (FTIR) spectroscopy of peptide amphiphiles. FTIR spectra of the E_2 PA, Ada-E_2 PA , CD-E_2 PA and the Host-Guest PA Complex.

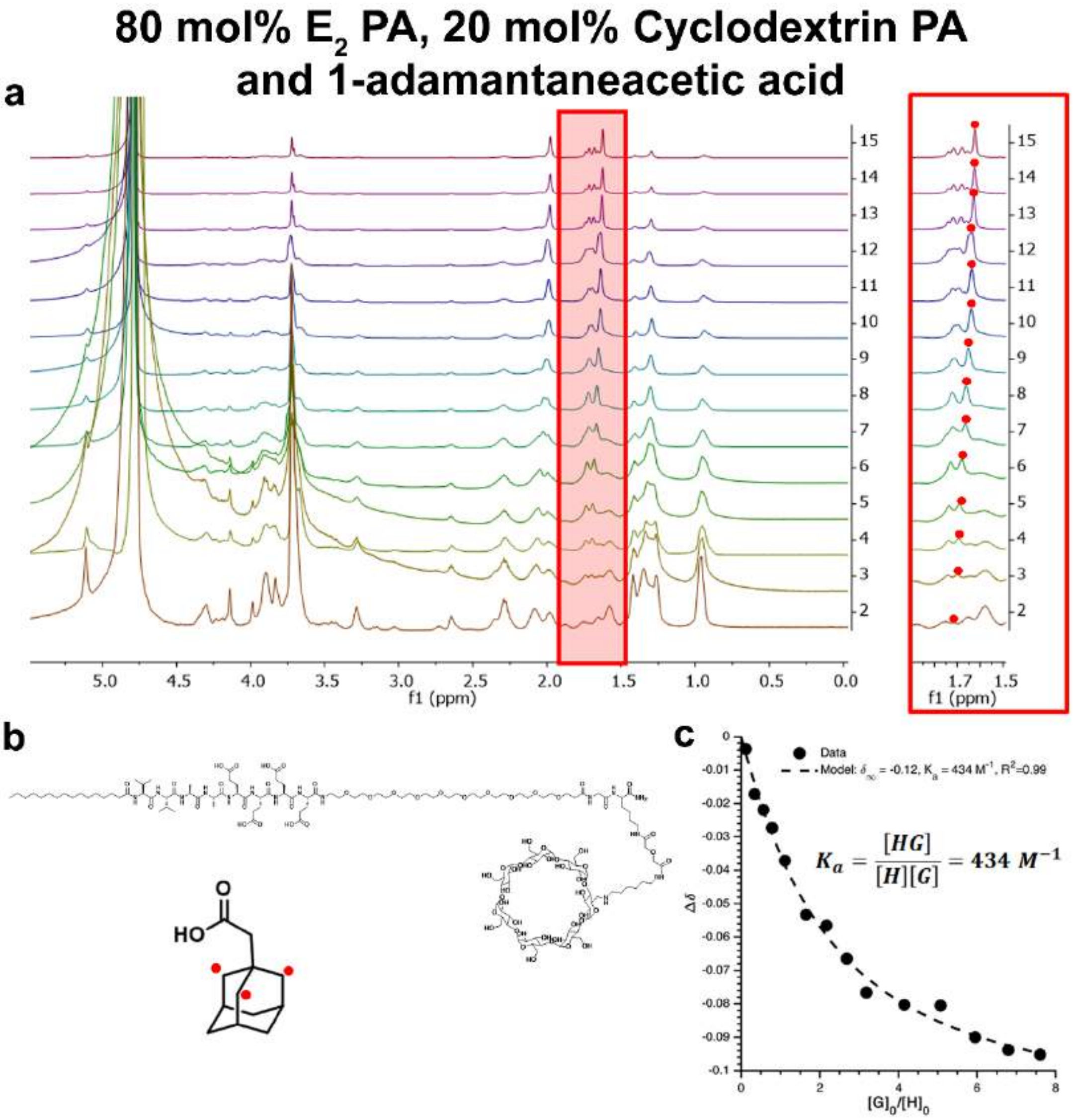

Figure S7. Determination of CD-E$_2$ PA and 1-adamantaneacetic acid binding constant. (a) Representative ^{1}H-NMR spectra for the titration between CD-E$_2$ PA and 1-adamantaneacetic acid, with the concentration of 1-adamantaneacetic acid increasing from the bottom to the top of the plot. The inset denotes the region of the spectrum used to calculate the binding constant. (b) Chemical structures of CD PA and 1-adamantaneacetic acid. The proton on 1-adamantaneacetic acid that was used to calculate the binding constant is labeled in red. (c) The binding isotherm for the titration experiment fit to a standard 1:1 host-guest binding model showing an association constant (K_a) of 434 M^{-1}.

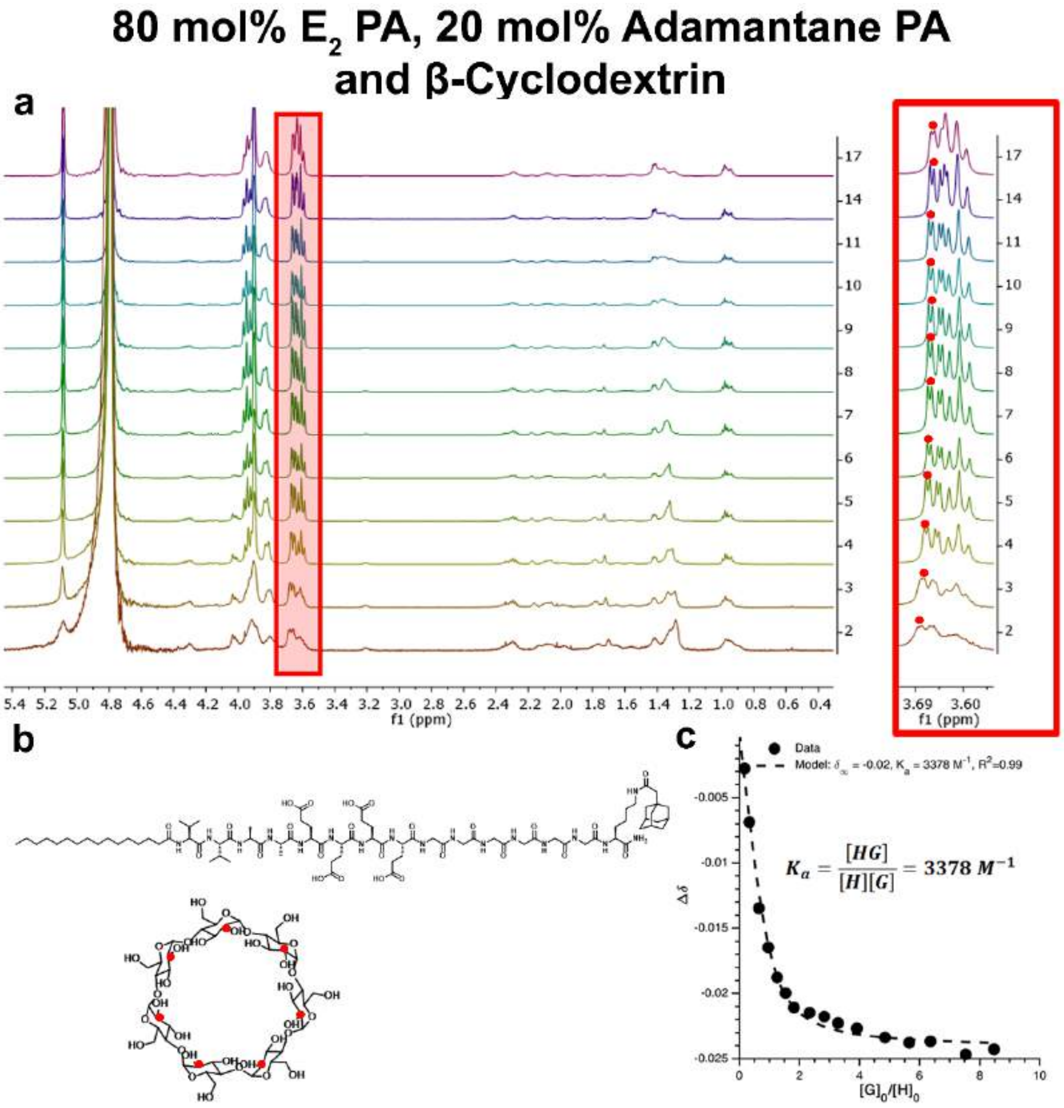

Figure S8. Determination of Ada-E2 PA and β-cyclodextrin binding constant. (a) Representative ^{1}H-NMR spectra for the titration between Ada-E_2 PA and β-cyclodextrin, with the concentration of β-cyclodextrin increasing from the bottom to the top of the plot. The inset denotes the region of the spectrum used to calculate the binding constant. (b) Chemical structures of Ada PA and β-cyclodextrin. The proton on β-cyclodextrin that was used to calculate the binding constant is labeled in red. (c) The binding isotherm for the titration experiment fit to a standard 1:1 host-guest binding model showing an association constant (K_a) of 3378 M^{-1}.

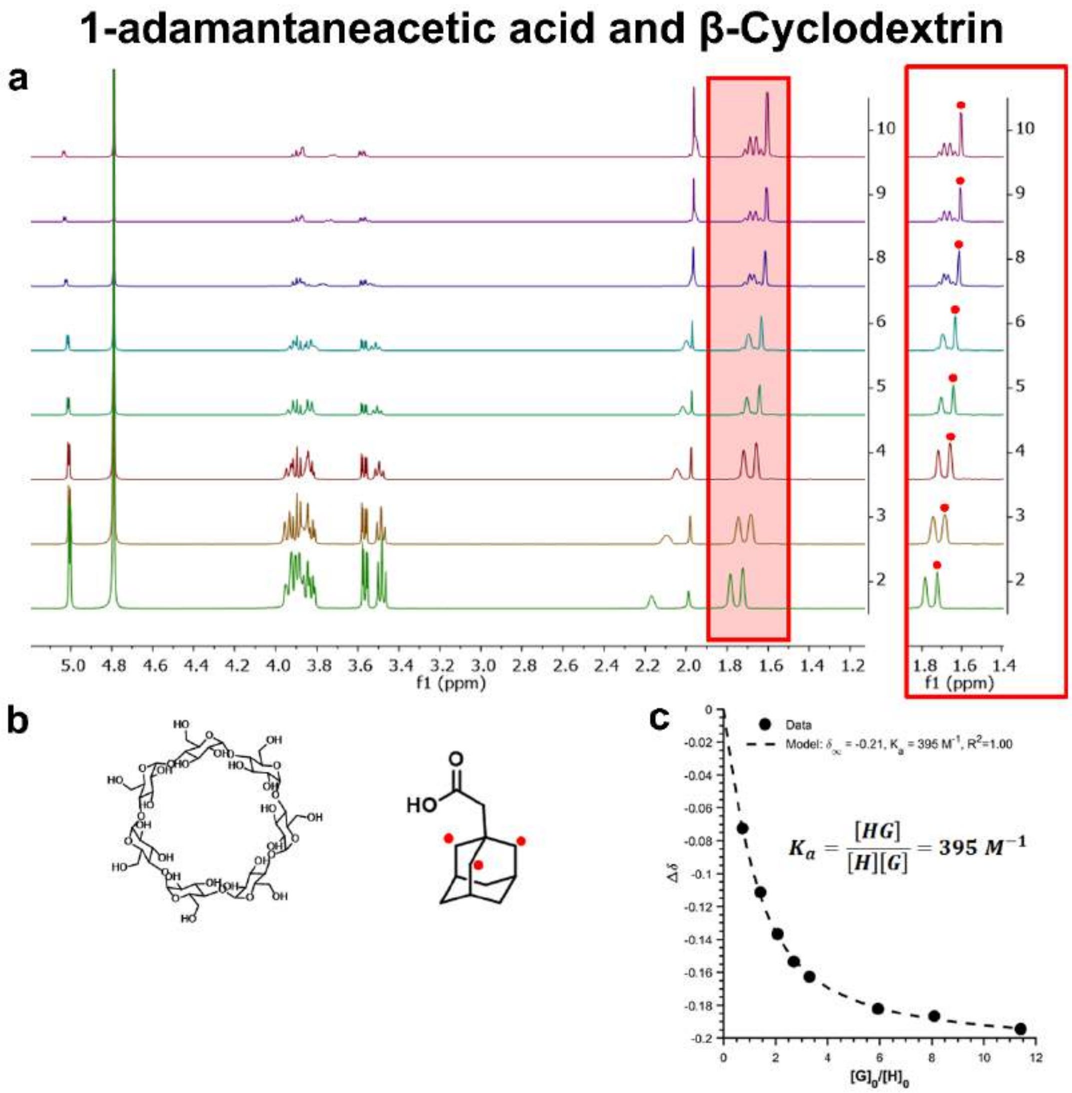

Figure S9. Determination of β-cyclodextrin and 1-adamantaneacetic acid binding constant. (a) Representative ^{1}H-NMR spectra for the titration between β-cyclodextrin and 1-adamantaneacetic acid, with the concentration of 1-adamantaneacetic acid increasing from the bottom to the top of the plot. The inset denotes the region of the spectrum used to calculate the binding constant. (b) Chemical structures of β-cyclodextrin and 1-adamantaneacetic acid. The proton on 1-adamantaneacetic acid that was used to calculate the binding constant is labeled in red. (c) The binding isotherm for the titration experiment fit to a standard 1:1 host-guest binding model showing an association constant (K_a) of 395 M^{-1}.

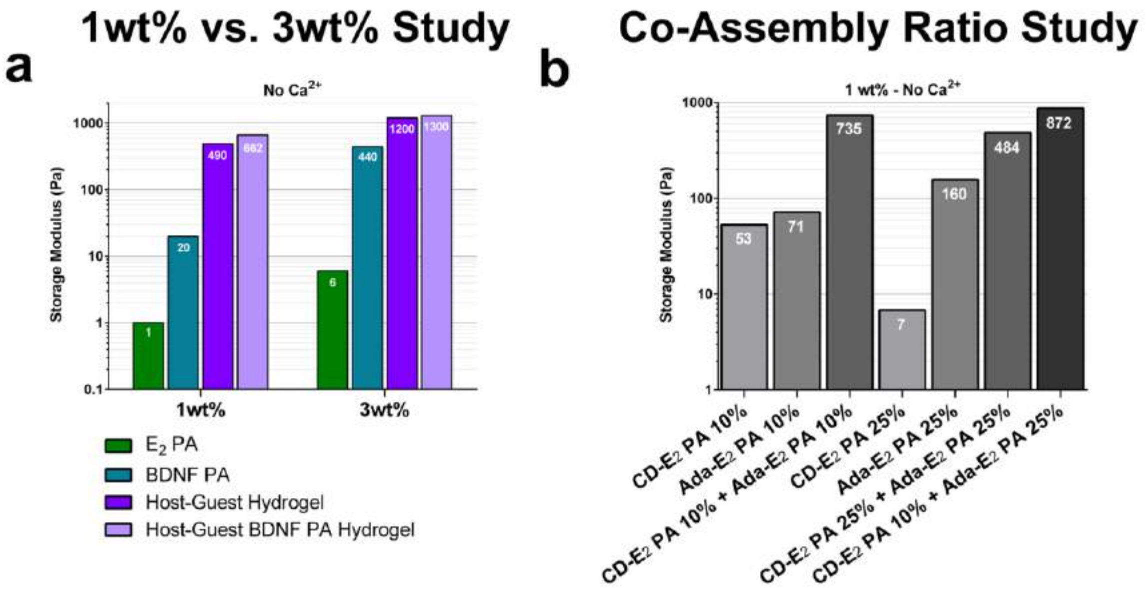

Figure S10. Comparisons of storage moduli for different hydrogel conditions (a) Comparison of storage moduli for 1 wt% versus 3 wt% hydrogels. (b) Comparison of different stoichiometric ratios of the Ada-PA relative to the CD-PA in the host-guest hydrogels at 1 wt% (n=1).

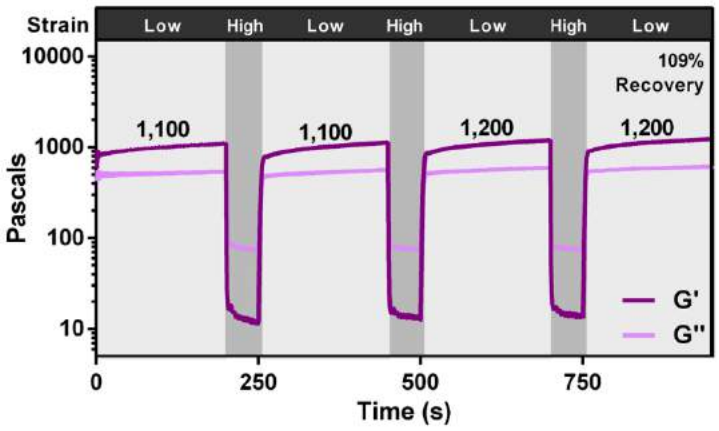

Figure S11. Rheological Recovery Test of the superstructured mixture. Storage (G') and loss modulus (G'') of the superstructured mixture as it was exposed to high and low shear intervals over time. With G' of 1,100 Pa before high strain and 1,200 Pa after 3 intervals of 50% strain with a 109% recovery.

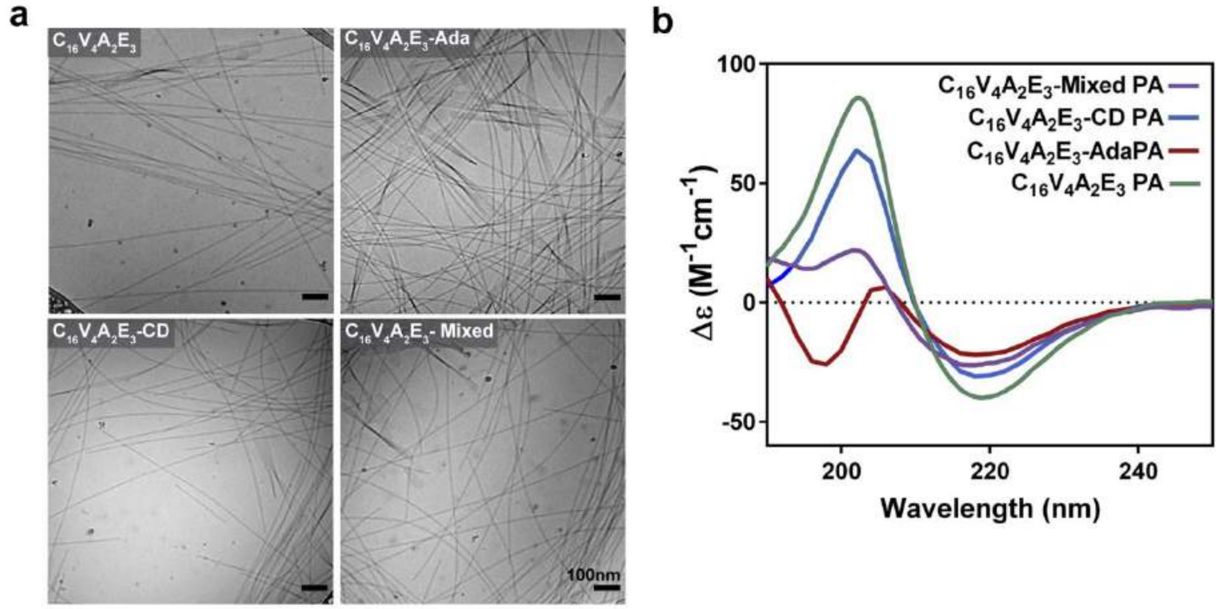

Figure S12. Fiber analysis of V_4-modified host-guest PAs. (a) Cryo-TEM of $C_{16}V_4A_2E_3$, $C_{16}V_4A_2E_3$-Ada, $C_{16}V_4A_2E_3$-CD, and $C_{16}V_4A_2E_3$-Mixed. (b) Circular dichroism spectra of samples in part (a).

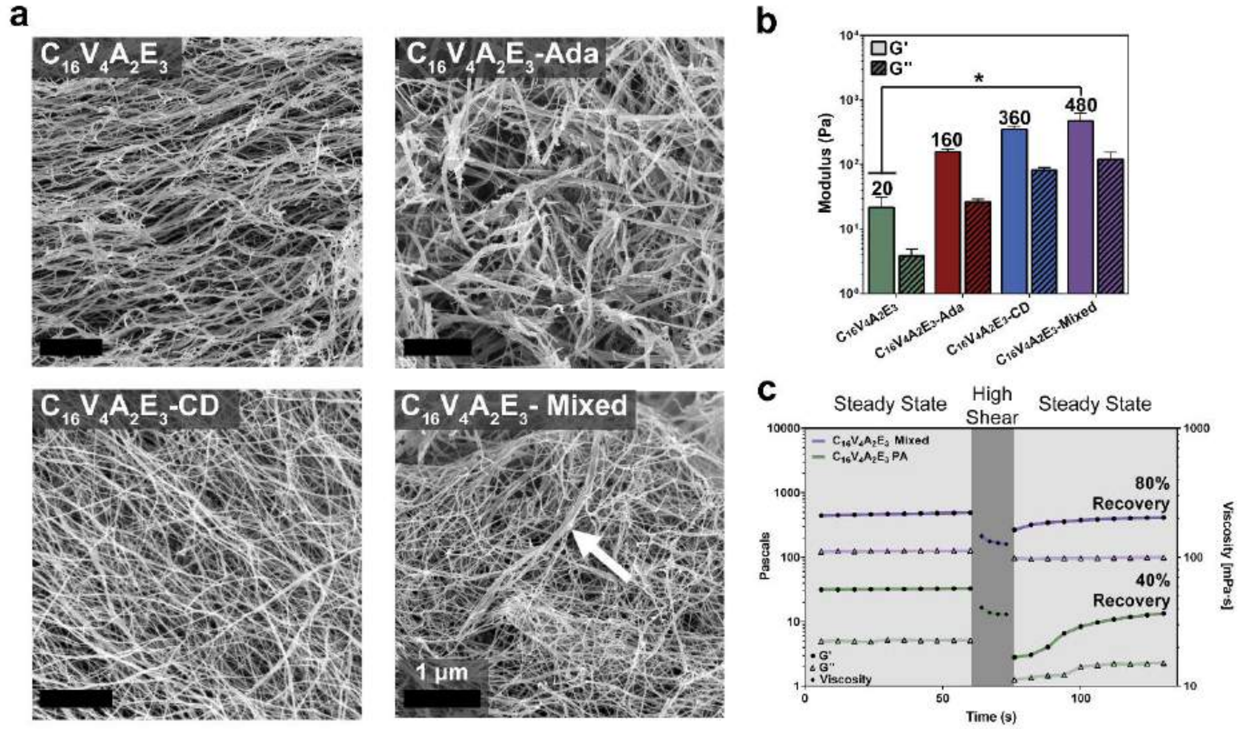

Figure S13. Macroscopic characterization of V_4-modified host-guest PAs. (a) SEM micrographs of $C_{16}V_4A_2E_3$, $C_{16}V_4A_2E_3$-Ada, $C_{16}V_4A_2E_3$-CD, and $C_{16}V_4A_2E_3$-Mixed. (b) Storage (G') and loss (G'') moduli of the samples described in part (a). (c) Thixotropy study showing storage modulus (G'), loss modulus (G'') and viscosity for the $C_{16}V_4A_2E_3$ and $C_{16}V_4A_2E_3$-Mixed PAs under three consecutive intervals of steady state, high shear, and a resting state. *P<0.1, LSD test (b) (n = 3).

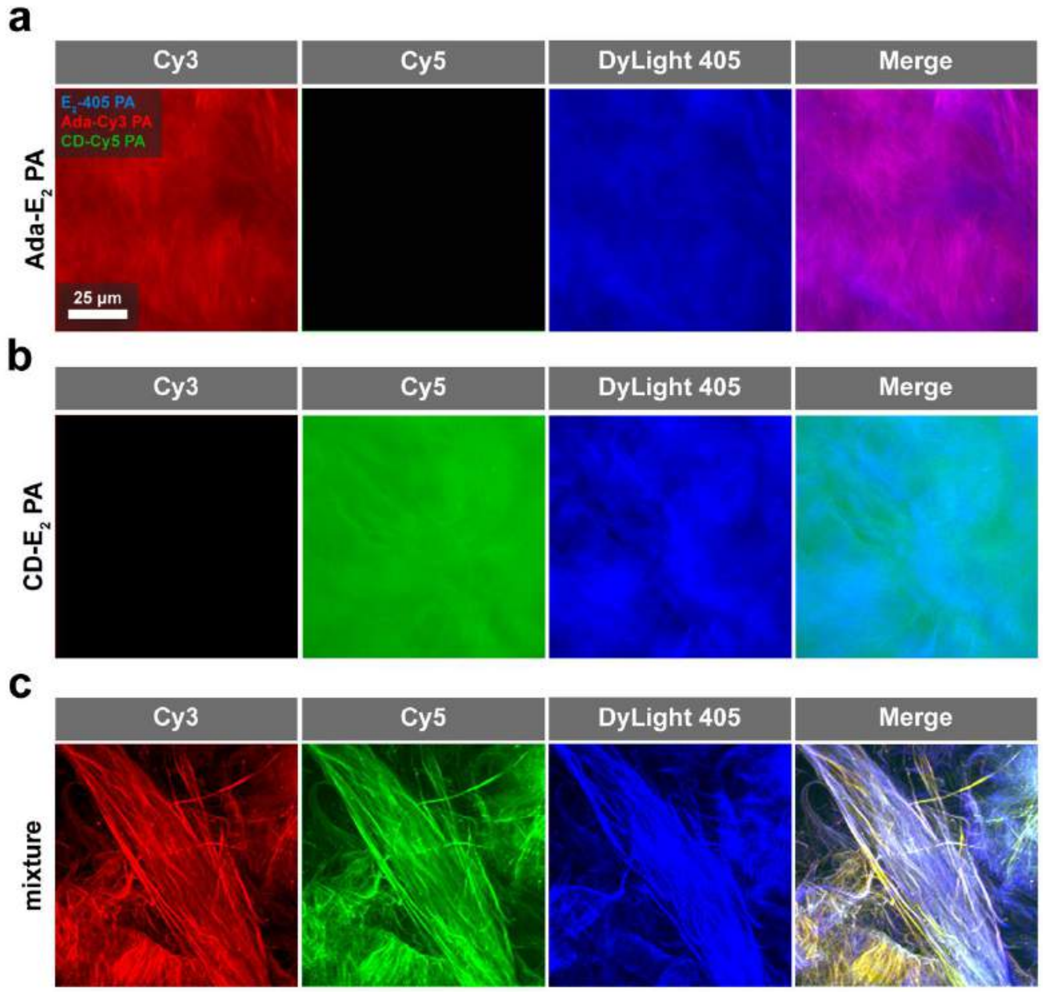

Figure S14. Split channels of the Ada-E_2 PA, CD-E_2 PA and superstructured mixture. Confocal Micrograph z-stacks split into individual channels (Left to right): Cy3 functionalized adamantane PA (Red), Cy5 functionalized cyclodextrin PA (Green), and DyLight 405 functionalized E_2 PA (Blue) of the (a) Ada-E_2 PA , (b) CD-E_2 PA, and (c) the superstructured host-guest hydrogel.

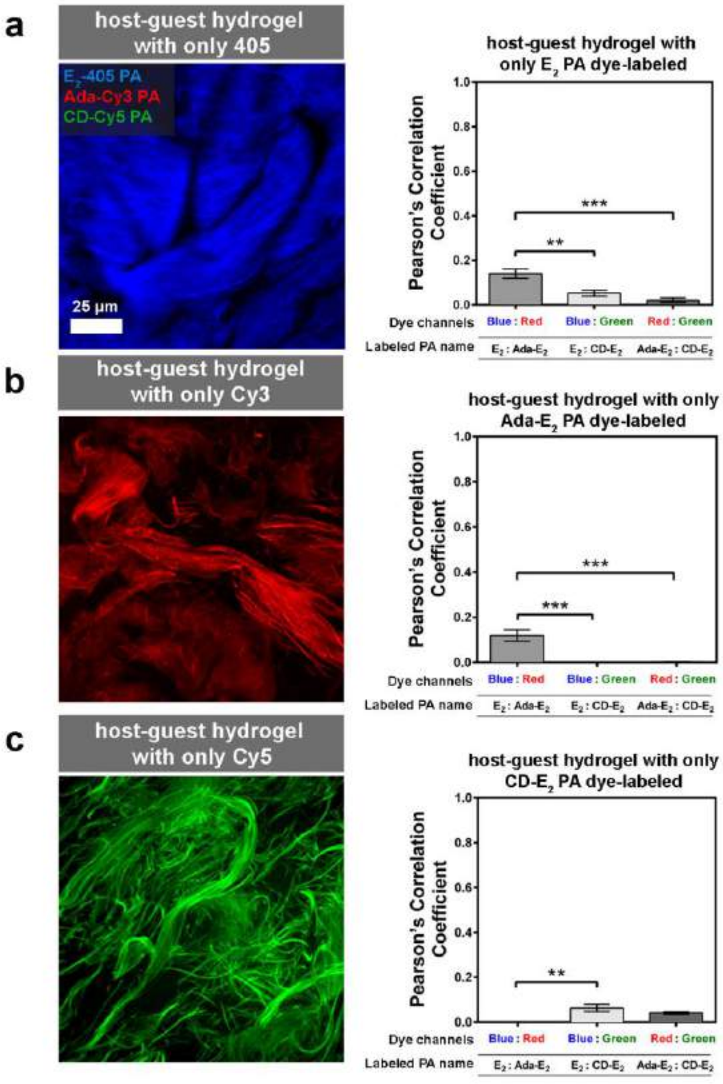

Figure S15. Pearson's correlation analysis of single fluorescent labelled control samples. Comparison of Pearson's correlation coefficients between fluorescent channels of images with only (a) the E_2 PA dye-labeled (405 only), (b) the Ada-E_2 PA dye-labeled (Cy3 only) and (c) the CD-E_2 PA dye-labeled (Cy5 only). Data displayed as mean ± SEM, with significance assessed with a one-way ANOVA followed by a Bonferroni post-hoc test, **P<0.01, ***P<0.001.

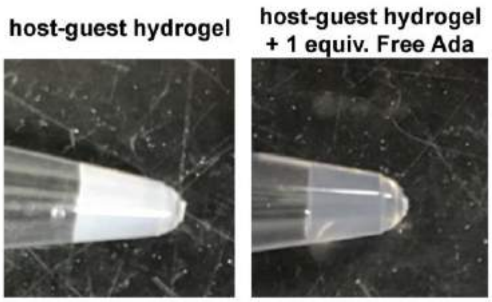

Figure S16. Photographs of superstructured host-guest hydrogel disassembly with free adamantane. Photographs of the superstructured host-guest hydrogel before (left) and after (right) the addition of 1 equivalent (equiv.) free adamantane (Free Ada).

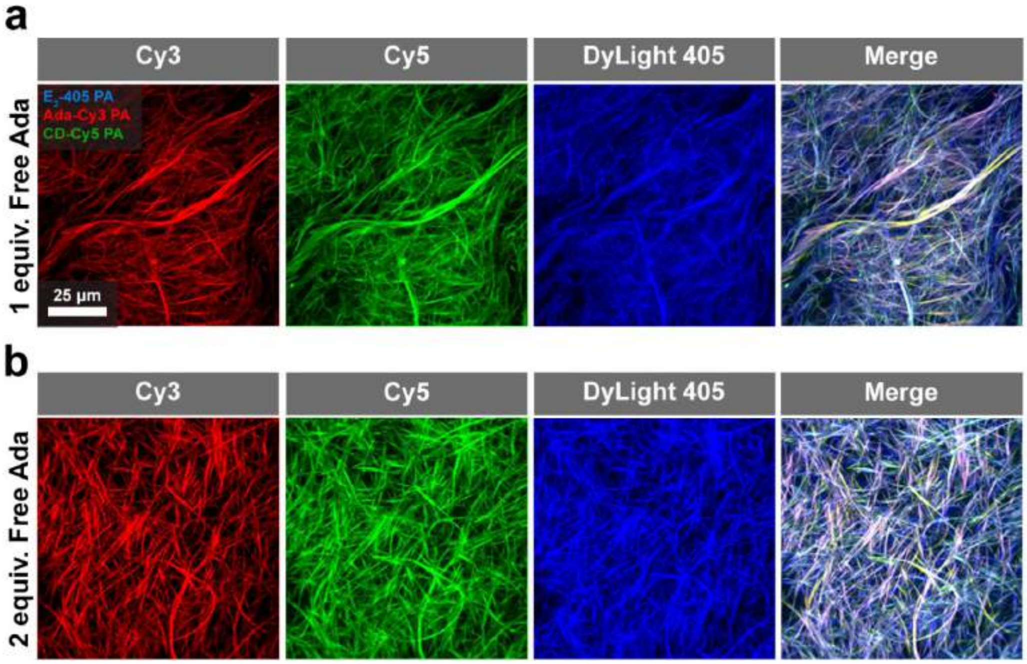

Figure S17. Fluorescent images of the superstructured host-guest hydrogel + 1 equivalent free adamantane and 2 equivalents free adamantane. Confocal micrograph z-stacks split into individual channels (Left to right): Cy3 functionalized adamantane PA (Red), Cy5 functionalized cyclodextrin PA (Green), and DyLight 405 functionalized E_2 PA (Blue) of the (a) superstructured host-guest hydrogel + 1 equiv free adamantane (Free Ada) and (b) superstructured host-guest hydrogel + 2 equiv free adamantane.

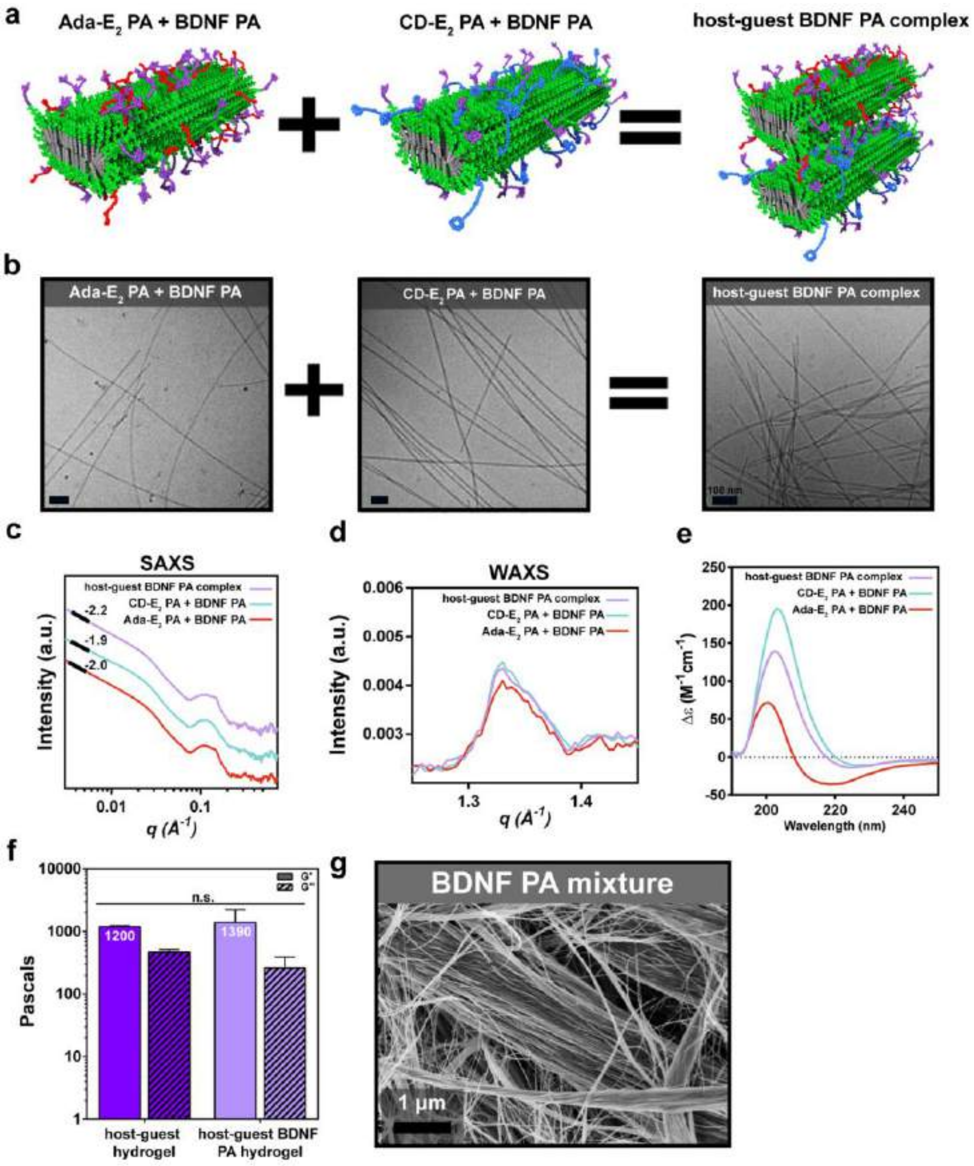

Figure S18. Characterization of the host-guest PA system with the incorporation of the BDNF PA. (a) Schematic representation of the inclusion of the BDNF mimetic PA into the adamantane-cyclodextrin superstructure forming system. (b) Cryo-TEM, (c) SAXS, (d) WAXS and (e) circular dichroism spectroscopy characterization of the Ada-E_2 PA + BDNF PA, CD-E_2 PA + BDNF PA and the BDNF superstructured mixture. (f) Rheology assessment of the host-guest BDNF PA hydrogel compared to the host-guest hydrogel without BDNF PA. (g) SEM micrograph of the BDNF PA mixture. (f) (n = 2).

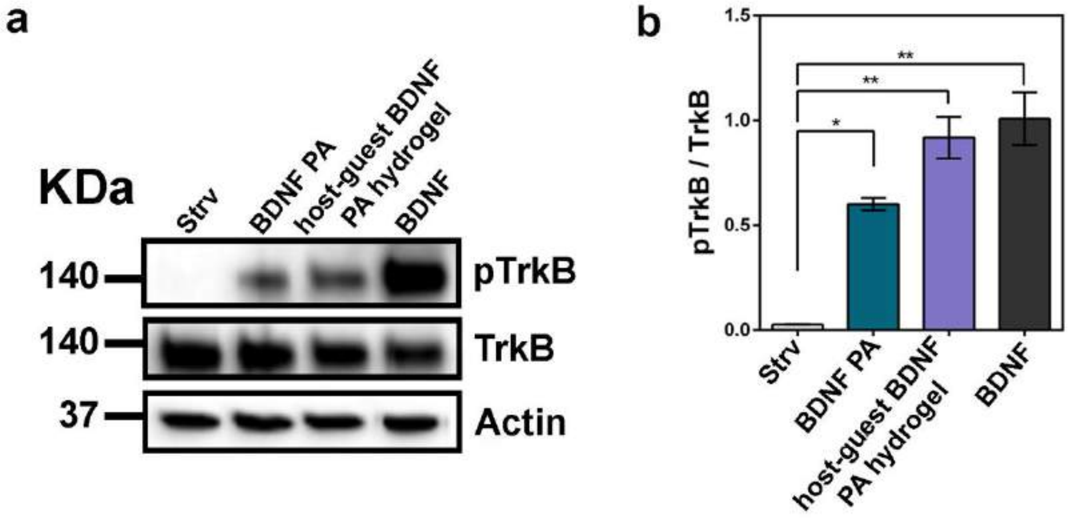

Figure S19. TrkB receptor activation of primary cortical neurons treated with BDNF superstructure. (a) Replicate of western blot of phosphorylated TrkB (p-TrkB), TrkB, and actin in neuronal cells exposed to starvation conditions (Strv), BDNF PA, host-guest BDNF PA superstructured material (host-guest BDNF), and BDNF protein (BDNF) *in vitro*. (b) Densitometry analysis of the western blot shown in (a). *P <0.05, and **P<0.01, LSD test (b) (n=3).

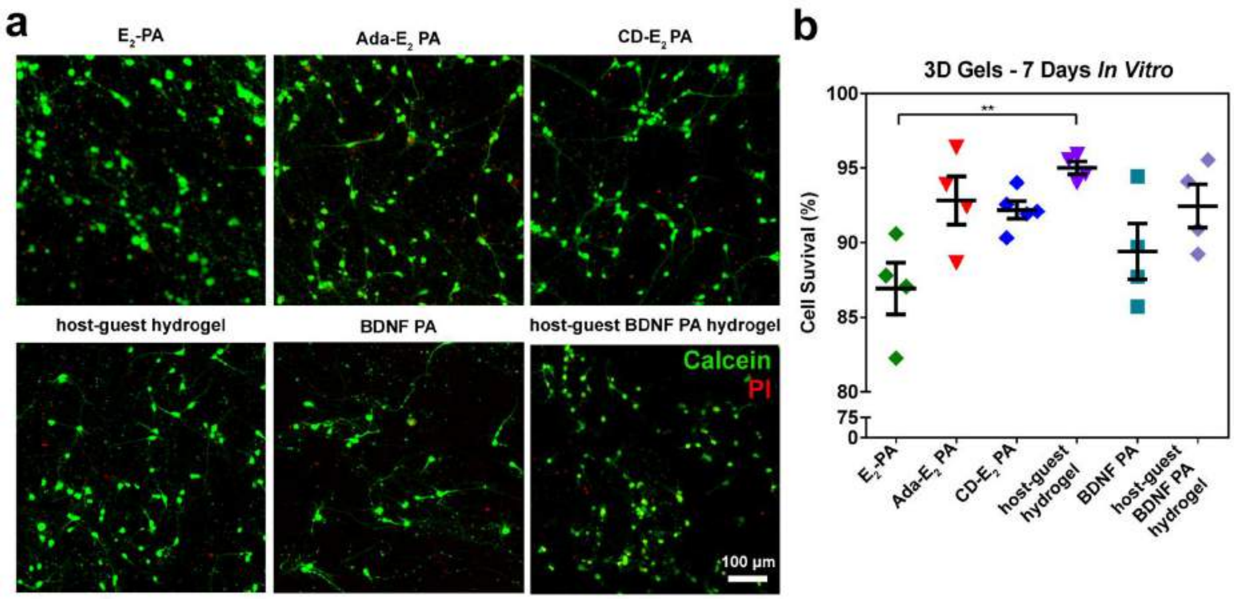

Figure S20. Live-Dead Analysis of embryonic primary cortical neurons seeded on 3D gels. (a) Confocal micrographs of cells cultured on 3D gels of the E_2-PA, Ada-E_2 PA, CD-E_2 PA, host-guest hydrogel, BDNF PA, and host-guest BDNF PA hydrogel stained with calcein (Green) and propidium iodide (Red) for 7 days *in vitro*. (b) Percent cell survival quantification of images in (a) (values normalized to total number of cells). **P<0.01, LSD test (b) (n = 4).

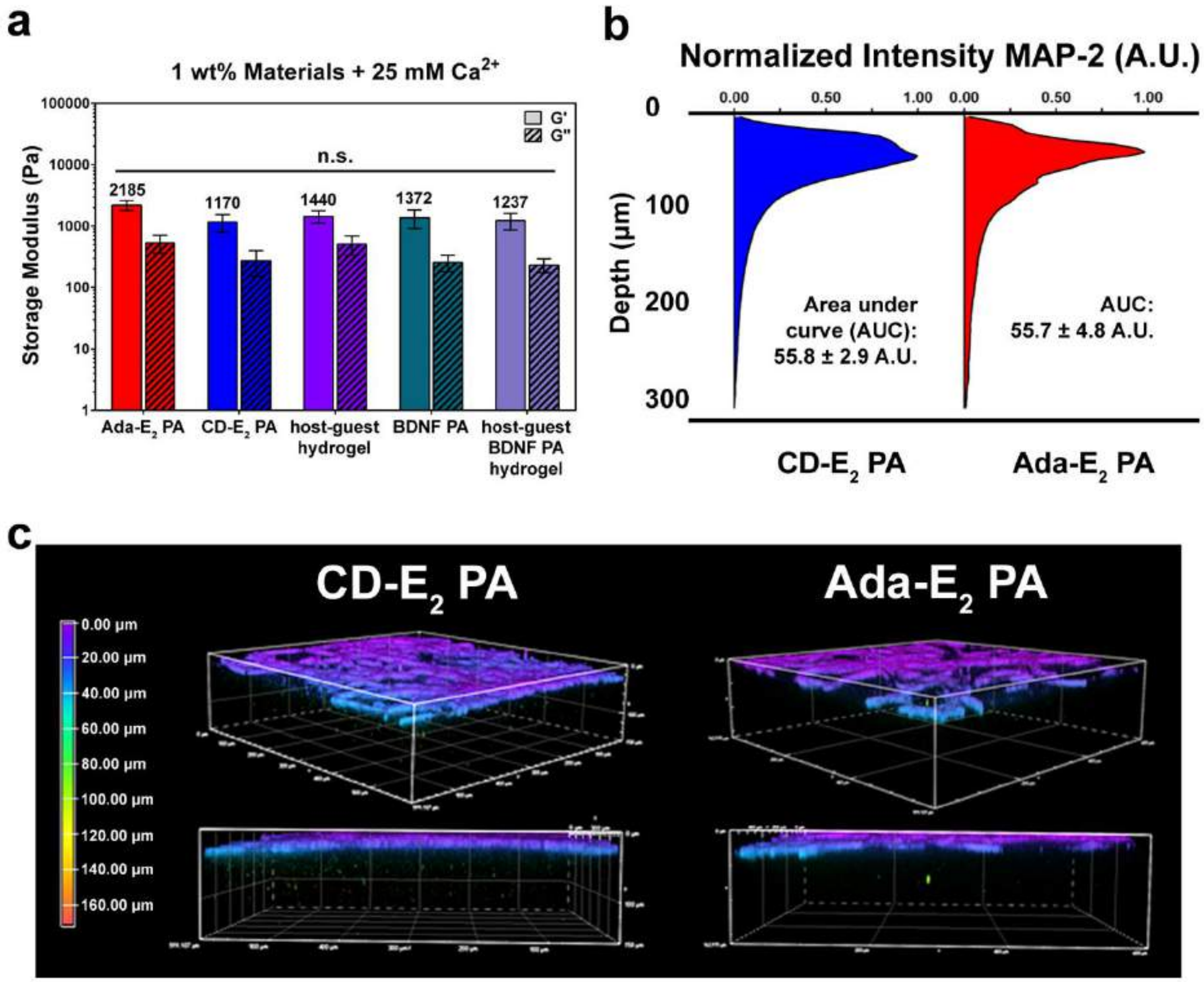

Figure S21. Rheological properties of PA scaffolds and infiltration of cortical neurons in PA scaffolds. (a) G' and G'' for Ada-E_2 PA, CD-E_2 PA, host-guest hydrogel, BDNF PA, and host-guest BDNF PA hydrogel using *in vitro* conditions of 1 wt% material and 25 mM Ca^{2+}. (b) Normalized average intensity of MAP-2 and pixel depth analysis of cell infiltration of cortical neurons on gels of the CD-E_2 PA, and the Ada-E_2 PA after 7 days *in vitro*. Area under the curve (AUC) displayed with the standard deviation, in arbitrary units (A.U.), for each gel type. (c) Depth-coded z-stack reconstructions showing cell infiltration on the CD-E_2 PA and Ada-E_2 PA gels after 7 days *in vitro*. (a) (n = 3), (b) (n = 3).

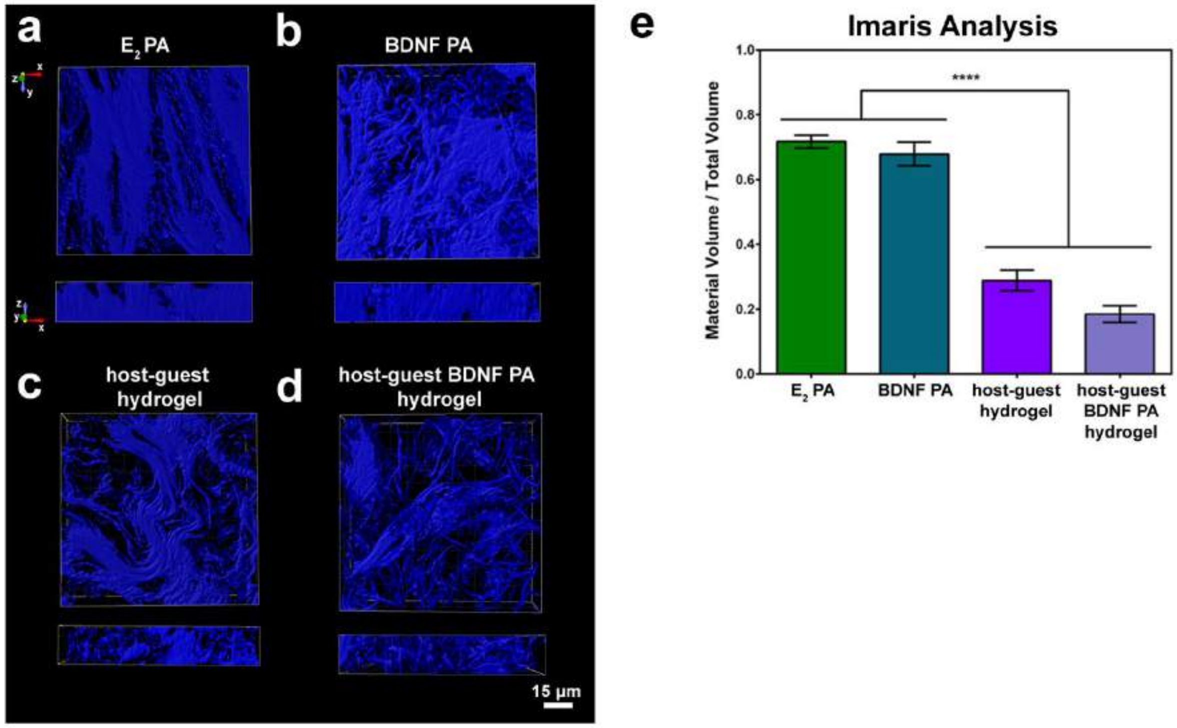

Figure S22. Shadow projection volume analysis of PA scaffolds. Shadow projections of PA scaffolds with E_2-405 dye labeled PA material to visualize material volume of the (a) E_2 PA (b) BDNF PA, (c) host-guest hydrogel and (d) host-guest BDNF PA hydrogel. (e) Quantification of the ratio of material volume to total scaffold volume for the three conditions. ****P<0.0001, LSD test (e) (n = 3).

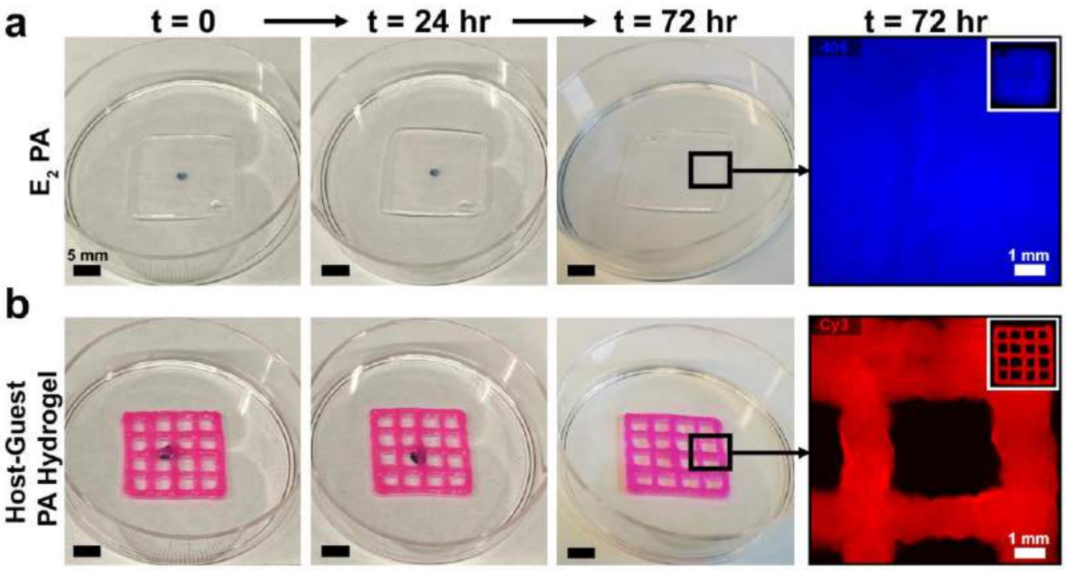

Figure S23. Host-guest hydrogel macroporous scaffold. Each material was extruded into a grid design to form a 20 x 20 mm macroporous scaffold. Images were taken directly after extrusion, at 24, and 72 hours (including fluorescence imaging) following extrusion to study if the materials were able to hold their shape. (a) The E_2 PA (E_2 PA + E_2-405 PA, prepared at 3 wt% and annealed) lost the definition of the structure during extrusion while (b) the host-guest PA hydrogel (CD-E_2 PA and Ada-E_2 PA + Ada-E_2-Cy3 PA, 3 wt% and annealed separately before being mixed 1:1 to create the host-guest PA hydrogel) exhibited a self-standing macroporous structure at t = 0, t = 24, and t = 72 hrs.

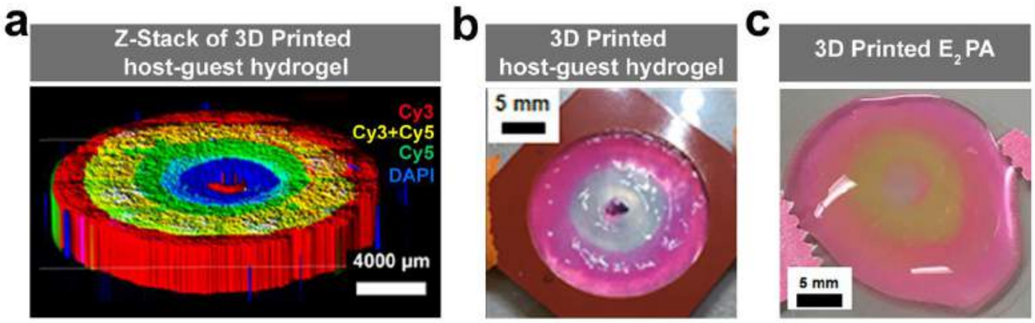

Figure S24. 3D printed concentric circles of the host-guest hydrogel. (a) Ti2 Widefield micrograph and 3D reconstruction of the 3D printed host-guest hydrogel pictured in Figure 4c. Labeled with fluorophores listed from outside in; Cy3, Cy3 + Cy5, Cy5, DAPI, Cy3 and (b) photograph of the 3D printed host-guest hydrogel (c) Photograph of E_2 PA liquid ink printed in concentric circles taken immediately after printing.

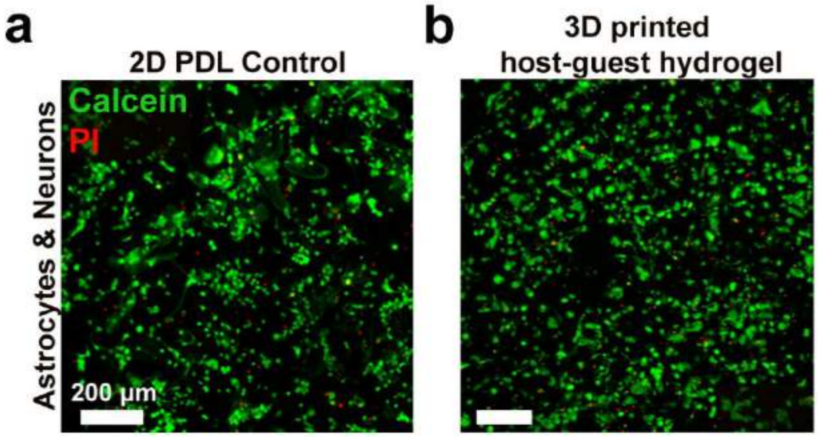

Figure S25. Live-Dead assay images. Confocal micrograph of primary cortical neurons and astrocytes stained for calcein (live marker, green) and propidium iodide (dead marker, red) of cells on a (a) 2D PDL control and (b) within the 3D printed host-guest hydrogel, both for 3 days *in vitro*.

4. Spectroscopic Data

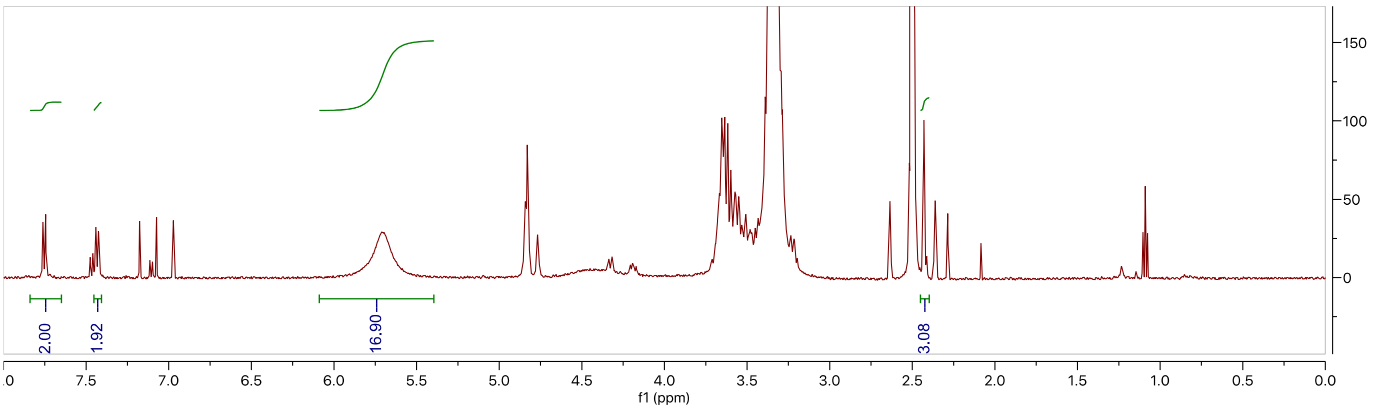

4.1. ^{1}H NMR spectrum of 6-*O*-monotosyl-6-deoxy-β-cyclodextrin (CD-tos).

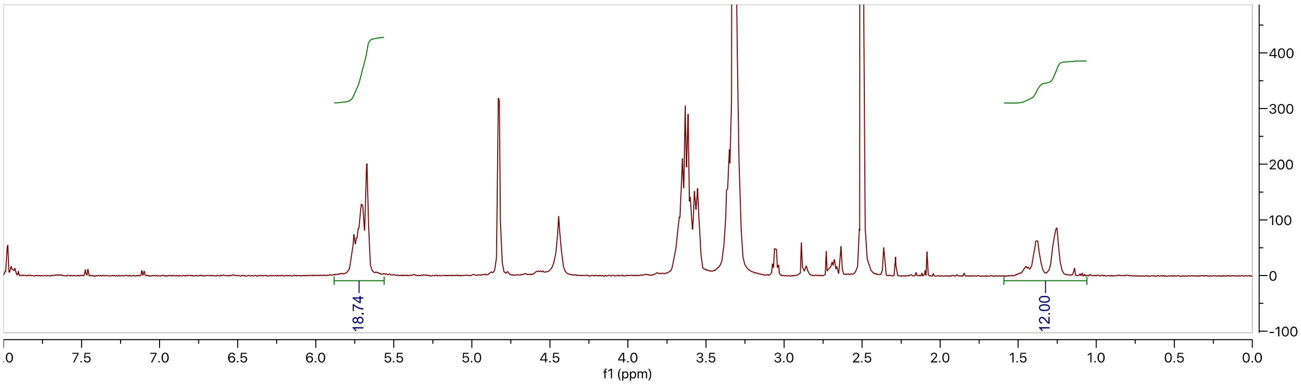

4.2. ^{1}H NMR spectrum of 6-(6-aminohexyl)amino-6-deoxy-β-cyclodextrin (CD-HDA).

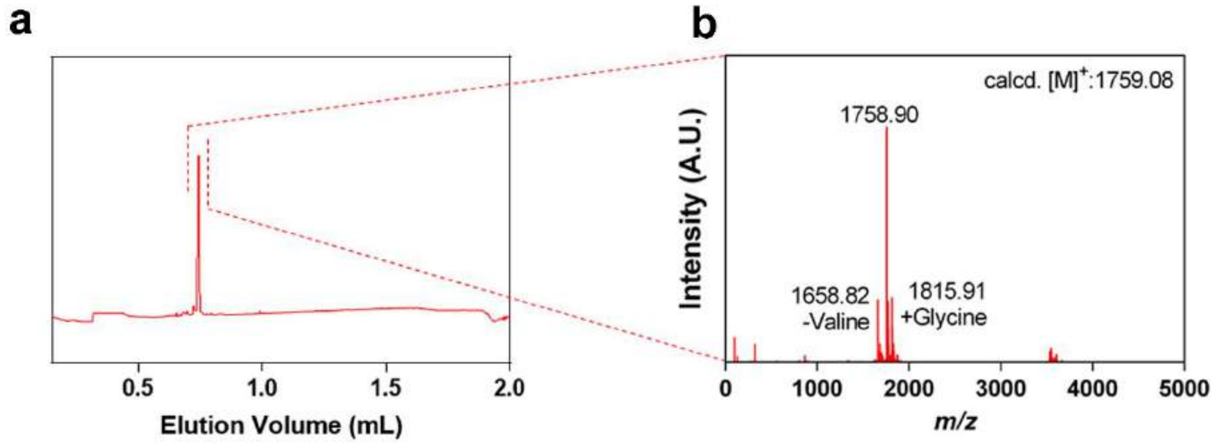

4.3. LCMS of the adamantane PA. (a) LCMS trace of adamantane PA [adamantane PA] = 1 mg/mL, loading solvent; H_20 with 0.1% NH_4OH (v/v), eluent; H_2O-CH_3CN gradient containing 0.1% HCOOH (v/v), column; Phenomenex Gemini 5 µm C18 110 Å LC column 150 x 1 mm. (b) ESI-mass spectra of adamantane PA. Elution volume 0.73 -0.75 mL.

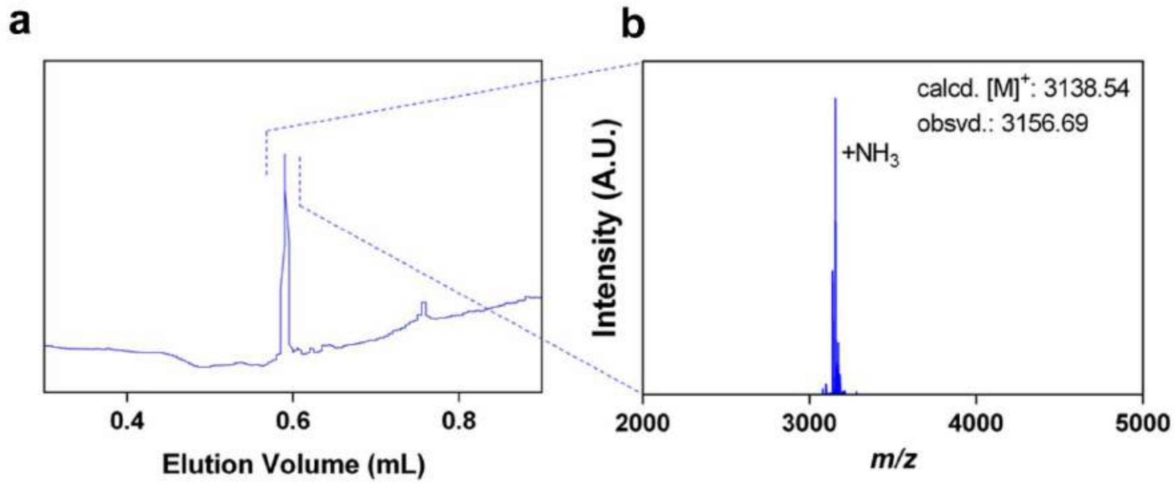

4.4. LCMS of the cyclodextrin PA. (a) LCMS trace of cyclodextrin PA [cyclodextrin PA] = 1 mg/mL, loading solvent; H_20 with 0.1% NH_4OH (v/v), eluent; H_2O-CH_3CN gradient containing 0.1% HCOOH (v/v), column; Phenomenex Gemini 5 µm C18 110 Å LC column 150 x 1 mm. (b) ESI-mass spectra of Cyclodextrin PA. Elution volume 0.595-0.605 mL.

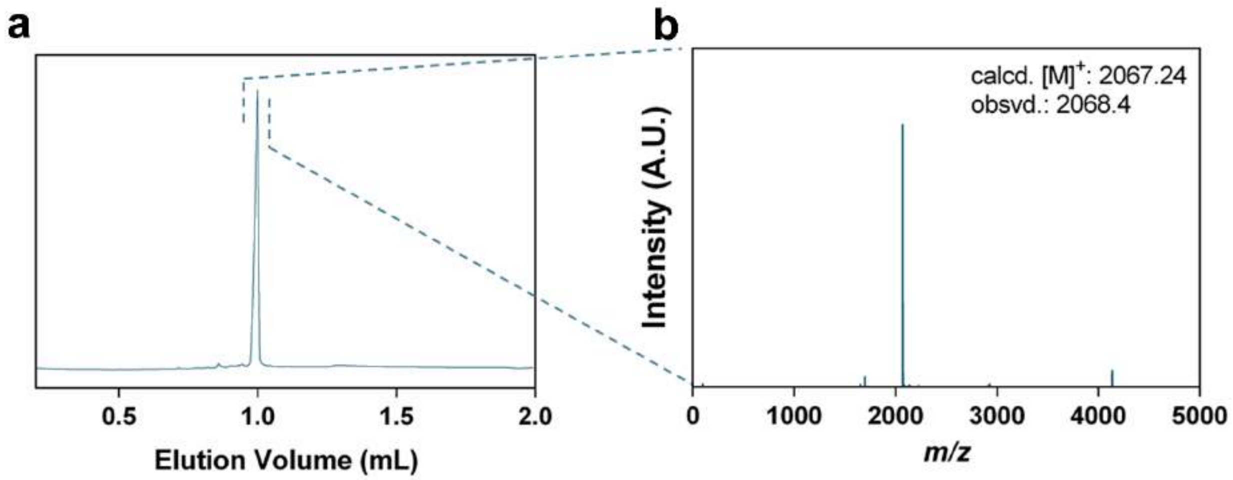

4.5. LCMS of the BDNF PA. (a) LCMS trace of BDNF PA [BDNF PA] = 1 mg/mL, loading solvent; H_20 with 0.1% NH_4OH (v/v), eluent; H_2O-CH_3CN gradient containing 0.1% HCOOH (v/v), column; Phenomenex Gemini 5 µm C18 110 Å LC column 150 x 1 mm. (b) ESI-mass spectra of BDNF PA. Elution volume 0.9731-1.0127 mL.

5. References

1. Loebel, C.; Rodell, C. B.; Chen, M. H.; Burdick, J. A., Shear-thinning and self-healing hydrogels as injectable therapeutics and for 3D-printing. *Nat Protoc* **2017,** *12* (8), 1521-1541.
2. Edelbrock, A. N.; Àlvarez, Z.; Simkin, D.; Fyrner, T.; Chin, S. M.; Sato, K.; Kiskinis, E.; Stupp, S. I., Supramolecular Nanostructure Activates TrkB Receptor Signaling of Neuronal Cells by Mimicking Brain-Derived Neurotrophic Factor. *Nano Lett* **2018,** *18* (10), 6237-6247.
3. Bolte, S.; Cordelieres, F. P., A guided tour into subcellular colocalization analysis in light microscopy. *J Microsc-Oxford* **2006,** *224*, 213-232.
4. Ortega, J. A.; Alcántara, S., BDNF/MAPK/ERK-induced BMP7 expression in the developing cerebral cortex induces premature radial glia differentiation and impairs neuronal migration. *Cereb Cortex* **2010,** *20* (9), 2132-44.
5. Álvarez, Z.; Mateos-Timoneda, M. A.; Hyroššová, P.; Castaño, O.; Planell, J. A.; Perales, J. C.; Engel, E.; Alcántara, S., The effect of the composition of PLA films and lactate release on glial and neuronal maturation and the maintenance of the neuronal progenitor niche. *Biomaterials* **2013,** *34* (9), 2221-33.
6. Schindelin, J.; Arganda-Carreras, I.; Frise, E.; Kaynig, V.; Longair, M.; Pietzsch, T.; Preibisch, S.; Rueden, C.; Saalfeld, S.; Schmid, B.; Tinevez, J. Y.; White, D. J.; Hartenstein, V.; Eliceiri, K.; Tomancak, P.; Cardona, A., Fiji: an open-source platform for biological-image analysis. *Nat Methods* **2012,** *9* (7), 676-82.
